# Lipidomic Alterations in the Cerebral Cortex and White Matter in Sporadic Alzheimer’s Disease

**DOI:** 10.14336/AD.2023.0217

**Published:** 2023-10-01

**Authors:** Elia Obis, Joaquim Sol, Pol Andres-Benito, Meritxell Martín-Gari, Natàlia Mota-Martorell, José Daniel Galo-Licona, Gerard Piñol-Ripoll, Manuel Portero-Otin, Isidro Ferrer, Mariona Jové, Reinald Pamplona

**Affiliations:** ^1^Department of Experimental Medicine, Lleida University (UdL), Lleida Biomedical Research Institute (IRBLleida), Lleida, Spain.; ^2^Catalan Institute of Health (ICS), Lleida, Spain, Research Support Unit (USR), Fundació Institut Universitari per a la Recerca en Atenció Primària de Salut Jordi Gol i Gurina (IDIAP JGol), Lleida, Spain.; ^3^CIBERNED (Network Centre of Biomedical Research of Neurodegenerative Diseases), Institute of Health Carlos III, Ministry of Economy and Competitiveness, Madrid, Spain.; ^4^Bellvitge University Hospital-Bellvitge Biomedical Research Institute (IDIBELL), E-08907 Hospitalet de Llobregat, Barcelona, Spain.; ^5^Unitat Trastorns Cognitius, Clinical Neuroscience Research, Santa Maria University Hospital, IRBLleida, Lleida, Spain.; ^6^Department of Pathology and Experimental Therapeutics, University of Barcelona, L’Hospitalet de Llobregat, Barcelona, Spain.

**Keywords:** Alzheimer disease, white matter, grey matter, lipidomics, peroxisomal beta-oxidation, lipid peroxidation

## Abstract

Non-targeted LC-MS/MS-based lipidomic analysis was conducted in post-mortem human grey matter frontal cortex area 8 (GM) and white matter of the frontal lobe *centrum semi-ovale* (WM) to identify lipidome fingerprints in middle-aged individuals with no neurofibrillary tangles and senile plaques, and cases at progressive stages of sporadic Alzheimer’s disease (sAD). Complementary data were obtained using RT-qPCR and immunohistochemistry. The results showed that WM presents an adaptive lipid phenotype resistant to lipid peroxidation, characterized by a lower fatty acid unsaturation, peroxidizability index, and higher ether lipid content than the GM. Changes in the lipidomic profile are more marked in the WM than in GM in AD with disease progression. Four functional categories are associated with the different lipid classes affected in sAD: membrane structural composition, bioenergetics, antioxidant protection, and bioactive lipids, with deleterious consequences affecting both neurons and glial cells favoring disease progression.

## INTRODUCTION

The cerebral white matter (WM) during the brain aging shows a reduction of the total volume and progressive alteration of the structural integrity, manifested as diffuse myelin decrease and focal lesions that lead to impaired brain connectivity [1-10]. These alterations bring to a cognitive and neuropsychiatric detriment [11-14]. Modification in the number and characteristics of oligodendrocytes and oligodendroglial precursor cells, responsible for myelin homeostasis, occurs in aged non-human primates and humans [15].

Neuroimaging methods also reveal alterations in the WM in sporadic Alzheimer's disease (sAD), including reduced WM size, hyper-intensities, myelin and axon damage, and reduced connectivity, in association with cognitive impairment [2, 3, 5, 12, 16-26]. WM alterations precede the appearance of clinical symptoms [27], and further deteriorates with AD progression [17-19, 23, 25]. The cerebral WM's atrophy and demyelination are also pinpointed in post-mortem neuropathological studies [28-31]. Therefore, loss of WM integrity is considered a key component of sAD, thus contributing to neural disconnection, cognitive impairment, and dementia [11].

Lipids have favored the brain evolution to attain its structural and functional complexity [32-34]. The human brain is one of the tissues richest in lipid content [35], with the most extensive diversity of lipid classes (for instance, glycerolipids (GLs), glycerophospholipids (GPs), sphingolipids (SLs) and cholesterol), and lipid molecular species. The human brain also has a wide diversity of functional properties covering the structural and functional integrity of neuronal and glial cell membranes, the generation of lipid mediators, and the chemical reactivity of the acyl chains [36]. Adult human brain lipids undergo slow but progressive and significant region-specific modifications in their concentration and distribution during aging. Thus, total lipid content -including fatty acids, GPs (especially ether lipids), SLs, and cholesterol-decreases after age 50 [36]. Lipoxidation-derived protein damage also increases with age in a region-specific manner [37-54].

Brain lipidomic analyses in sAD have identified multiple disease-specific lipid alterations and lipid-derived molecular damage [36, 55-68]. These alterations include depletion of ether lipids and sulfatides, increased ceramides (Cer), and severe lipoxidative damage. Lipidomic modifications detected at the early stages of sAD aggravate the disease's progression. Nevertheless, described changes in sAD mainly refer to different regions of the grey matter (GM), whereas studies of lipid changes in the WM in sAD are minimal [69].

A seminal study described differences in the lipid composition between human WM and GM through the lifespan [70]. In the adult brain, the total amount of lipids in the GM was 36-40% and 19-66% in the WM. The WM showed higher levels of sphingolipids (including sphingomyelins (SMs), cerebrosides, cerebrosides sulfatides, Cer, and cholesterol in comparison with the GM. No age-related WM changes were observed in total GPs, but glycerophosphatidylserines (PS) were increased, and glycerophosphatidylcholines (PCs) decreased [70].

This study was designed to assess lipid alterations separately in the GM of the frontal cortex area 8 and WM of the frontal lobe’s *centrum semi-ovale* in aging and sAD at different stages of progression. Cases with clinical and pathological co-morbidities were not included in the study. LC-MS/MS platform and gas chromatography were used for the lipidomics study. mRNA expression and proteins involved in lipid metabolism were analyzed by RT-qPCR and immunohistochemistry, respectively. We aimed to identify lipidome differences between the GM and WM in the brain aging and sAD using novel high throughput mass spectrometry-based techniques combined with protein expression analysis involved in selected lipid metabolism pathways, demonstrating that sAD is associated with altered lipidome profiles.

## MATERIAL AND METHODS

### Selection of human samples

Post-mortem samples of fresh-frozen tissue from the frontal cortex area 8 and *centrum semi-ovale* of the frontal lobe were obtained from the Institute of Neuropathology HUB-ICO-IDIBELL Biobank, following the guidelines of Spanish legislation on this matter (Real Decreto 1716/2011), and the approval of the local ethics committee.

One hemisphere was immediately cut in coronal sections 1-cm thick and selected areas of the encephalon were rapidly dissected, frozen on metal plates over dry ice, placed in individual air-tight plastic bags, and stored at -80°C until used for biochemical studies. The other hemisphere was fixed by immersion in 4% buffered formalin for three weeks for morphological studies. The neuropathological study was carried out on selected 4-μm-thick de-waxed paraffin sections of 20 representative regions. Sections were stained with hematoxylin and eosin, periodic acid-Schiff (PAS), and Klüver-Barrera, or processed for immunohistochemistry for β-amyloid, phospho-tau (clone AT8), α-synuclein, αB-crystallin, TDP-43, ubiquitin, p62, glial fibrillary acidic protein, CD68, and Iba1 [71]. The post-mortem delay varied from 1-hour and 30-minutes to 16-hours (Table 1). The brain pH at the autopsy was between 6.2 and 6.4, and the RNA integrity number (RIN) was higher than 6, thus ensuring the biological sample's quality [71-73].

sAD cases were categorized according to Braak and Braak [74] neurofibrillary tangle (NFT) and β-amyloid stages as ADI-II/0-A (n = 9, men: 5, women: 4); ADIII- IV/0-C (n = 9, men: 5, women: 5), and ADV-VI/B-C (n = 7, men: 4, women: 3) (Table 1). Cases with concomitant pathologies and co-morbidities were excluded, including age-related neurodegenerative diseases (tauopathies, Lewy body diseases, TDP-43 proteinopathy), hippocampal sclerosis; and those who had suffered from cerebrovascular disease, arterial hypertension, type II diabetes, hyperlipidemia, cardiac, hepatic, renal failure, and respiratory insufficiency. All selected cases were sporadic; familial AD was not included in this study. AD cases at stages I-II/0-A had no neurological symptoms; AD cases at stages III-IV/0-C had no neurological symptoms or were affected by mild cognitive impairment; AD cases at stages V-VI/B-C had severe cognitive impairment or dementia.

**Table 1:** Summary of middle-aged individuals without NFTs and SPs in any brain region (MA) and cases at different Braak stages of Alzheimer’s disease (AD) without co-morbidities and concomitant brain pathologies.

Case ID	Diagnosis	Sex	Age	PM delay
1	MA	F	46	14 h 05 min
2	MA	M	39	09 h 15 min
3	MA	M	55	05 h 40 min
4	MA	M	53	03 h 00 min
5	MA	F	82	11 h 00 min
6	MA	M	35	17 h 00 min
7	MA	F	54	06 h 45 min
8	MA	M	50	17 h 15 min
9	MA	M	57	03 h 00 min
10	MA	F	66	04 h 15 min
11	AD I/0	M	56	07 h 10 min
12	AD I/A	M	66	09 h 45 min
13	AD I/A	F	74	02 h 45 min
14	AD I/A	F	57	05 h 00 min
15	AD II/0	M	67	07 h 15 min
16	AD II/0	M	57	04 h 30 min
17	AD II/A	F	88	08 h 00 min
18	AD II/A	M	66	04 h 55 min
19	AD II/A	F	86	02 h 15 min
20	AD III/0	M	66	05 h 45 min
21	AD III/0	F	79	03 h 35 min
22	AD III/0	M	81	01 h 30 min
23	AD III/A	F	77	03 h 10 min
24	AD III/A	F	82	02 h 00 min
25	AD III/B	F	76	03 h 50 min
26	AD IV/A	F	80	02 h 45 min
27	AD IV/B	M	84	12 h 45 min
28	AD IV/C	F	81	05 h 00 min
29	AD V/0	F	74	05 h 30 min
30	AD V/B	M	86	04 h 15 min
31	AD V/B	M	73	04 h 30 min
32	AD V/C	M	77	16 h 00 min
33	AD V/C	F	85	16 h 15 min
34	AD V/C	F	72	09 h 30 min
35	AD V/C	M	85	03 h 45 min

M: male; F: female; PM: post-mortem delay; MA: middle-aged cases. NFTs: neurofibrillary tangle. SPs: senile plaques

The control group of middle-aged cases (MA) comprises 10 individuals, 4 female and 6 males. MA did not have clinical risk factors and co-morbidities mentioned in previous paragraphs; they did not have neurological or mental diseases, and the neuropathological study did not show abnormalities. This MA group must not be interpreted as an age-matched control group but as a control group of normal WM in MA individuals. The total number of MA and AD cases in this series is detailed in Table 1.

For lipidomic analyses the MA group for WM comprised 6 individuals (3 men and 3 women) whereas 4 for GM (3 women and 1 men). ADI-II/0-A group comprised 7 individuals (men: 3, women: 4); 5 individuals for ADIII- IV/0-C group (men: 1, women: 4), and 6 for ADV-VI/B-C (men: 4, women: 2).

### Fatty acid profiling

Fatty acyl groups were analyzed as methyl esters derivatives by gas chromatography as previously described [46]. For tissue homogenization, 50 mg of GM and WM was processed in a buffer containing 180 mM KCl, 5 mM MOPS, 2mM EDTA, 1 mM diethylenetriaminepentaacetic acid, and 1μM butylated hydroxytoluene. Tissue samples were randomized prior to lipid extraction. Quality control samples were included at a ratio of 1:5. Total lipids from samples were extracted into chloroform:methanol (2:1, v/v) in the presence of 0.01% (w/v) butylated hydroxytoluene. The chloroform phase was evaporated under nitrogen, and the fatty acyl groups were transesterified by incubation in 2.5mL of 5% (v/v) methanolic HCl at 75°C for 90 min. The resulting fatty acid methyl esters were extracted by adding 1mL of saturated NaCl solution and 2.5 mL of n-pentane. The n-pentane phase was separated and evaporated under N_2_. The residue was dissolved in 50µL of CS_2_, and 2µL was used for analysis. Separation was performed by a DBWAX capillary column (30m x 0.25mm x 0.20μm) in a GC System 7890A with a Series Injector 7683B and an FID detector (Agilent Technologies, Barcelona, Spain). The sample injection was in splitless mode. The injection port was maintained at 250°C, and the detector at 250°C. The program consisted of 5 min at 145°C, followed by 2°C/min to 245°C, and finally 245°C for 10 min, with a post-run at 250°C for 10 minutes. The total run time was 65 minutes, with a post-run time of 10 minutes. Identification of fatty acid methyl esters was made by comparison with authentic standards (Larodan Fine Chemicals, Malmö, Sweden) using specific software of data analysis for GC from Agilent (OpenLAB CDS Chem Station v. C.01.10; Agilent Technologies, Barcelona, Spain) and subsequent expert's revision and confirmation. Results are expressed as mol%.

The following fatty acyl indices were also calculated: saturated fatty acids (SFA); unsaturated fatty acids (UFA); monounsaturated fatty acids (MUFA); polyunsaturated fatty acids (PUFA) from n-3 and n-6 series (PUFAn-3 and PUFAn-6, respectively); and average chain length, ACL = [(Σ%Total_14_ x 14) + (Σ% Total_16_ ×16) + (Σ%Total_18_ ×18) + (Σ%Total_20_ ×20) + (Σ% Total_22_ ×22) + (Σ% Total_24_ ×24)]/100. The density of double-bonds in the membrane was calculated with the Double-Bond Index, DBI = [(1 × Σmol% monoenoic) + (2 × Σmol% dienoic) + (3 × Σmol% trienoic) + (4 × Σmol% tetraenoic) + (5 × Σmol% pentaenoic) + (6 × Σmol% hexaenoic)]. Membrane susceptibility to peroxidation was calculated with the Peroxidizability Index, PI (a) = [(0.025 × Σmol% monoenoic) + (1 × Σmol% dienoic) + (2 × Σmol% trienoic) + (4 × Σmol% tetraenoic) + (6 × Σmol% pentaenoic) + (8 × Σmol% hexaenoic)] [75], and PI (b) = [(0.015 × Σmol% monoenoic) + (1 × Σmol% dienoic) + (2 × Σmol% trienoic) + (3 × Σmol% tetraenoic) + (4 × Σmol% pentaenoic) + (5 × Σmol% hexaenoic)] [76, 77].

Elongase and desaturase activities were estimated from specific product/substrate ratios [78]: Elovl3(n-9) = 20:1n-9/18:1n-9; Elovl6 = 18:0/16:0; Elovl1-3-7a = 20:0/ 18:0; Elovl1-3-7b = 22:0/20:0; Elovl1-3-7c = 24:0/22:0; Elovl5(n-6) = 20:2n-6/18:2n-6; Elovl2-5 (n-6) = 22:4n-6/20:4n-6; Elovl 2-5(n-3) = 22:5n-3/20:5n-3, Elovl 2(n-3)= 24:5n-3/22:5n-3, Δ9(n-7) = 16:1n-7/16:0; Δ9(n-9) = 18:1n-9/18:0; Δ5(n-6) = 20:4n-6/20:3n-6; Δ6(n-3) (a) = 18:4n-3/18:3n-3; and Δ6(n-3) (b) = 24:6n-3/24:5n-3. Finally, the peroxisomal β-oxidation was estimated according to the 22:6n-3/24:6n-3 ratio.

### Non-targeted lipidomic analysis

*Sample preparation.* For the lipid extraction, 10μL of the homogenized tissue were mixed with 5μL of MiliQ water and 20μL of ice-cold methanol. Samples were vigorously shaken by vortexing for 2 min, and then 250μL of methyl tert-butyl ether (MTBE), containing internal lipid standards (see Supplementary Table 1), were added. Samples were immersed in a water bath (ATU Ultrasonidos, Valencia, Spain) with an ultrasound frequency and power of 40 kHz and 100 W, respectively, at 10°C for 30 min. Then, 25μL MiliQ water was added to the mixture, and the organic phase was separated by centrifugation (1,400 g) at 10°C for 10 min [79]. Lipid extracts in the upper phase were subjected to mass spectrometry. A pool of all lipid extracts was prepared and used as quality control. Internal isotopically labeled lipid standards for each class were used for signal normalization [80]. Stock solutions were prepared by dissolving lipid standards in MTBE at a concentration of 1mg/mL, and working solutions were diluted to 2.5μg/mL in MTBE.

*LC-MS analysis.* Lipid extracts were analyzed following a previously published method [81]. Lipid extracts were subjected to liquid chromatography-mass spectrometry using a UPLC 1290 series coupled to ESI-Q-TOF MS/MS 6545 (Agilent Technologies, Barcelona, Spain). The sample compartment of the UHPLC was refrigerated at 4°C, and for each sample, 10μL of lipid extract was applied onto a 1.8μm particle 100 × 2.1mm id Waters Acquity HSS T3 column (Waters, Milford, MA, USA) heated at 55°C. The flow rate was 400μL/min with solvent A composed of 10mM ammonium acetate in acetonitrile-water (40:60, v/v) and solvent B composed of 10mM ammonium acetate in acetonitrile-isopropanol (10:90, v/v). The gradient started at 40% of mobile phase B, reached 100% B in 10 min, and held for 2 min. Finally, the system was switched back to 40% of mobile phase B and was equilibrated for 3 min. Duplicate runs of the samples were performed to collect positive and negative electrospray-ionized lipid species in a TOF mode, operated in full-scan mode at 100 to 3000 m/z in an extended dynamic range (2GHz), using N_2_ as nebulizer gas (5L/min, 350°C). The capillary voltage was set at 3500V with a scan rate of one scan/s. Continuous infusion using a double spray with masses 121.050873, 922.009798 (positive ion mode) and 119.036320, 966.000725 (negative ion mode) was used for in-run calibration of the mass spectrometer [82].

*Lipidomic data pre-processing and annotation.* MassHunter Qualitative Analysis Software (Agilent Technologies, Barcelona, Spain) was used to obtain the molecular features of the samples, representing different co-migrating ionic species of a given molecular entity using the Molecular Feature Extractor algorithm (Agilent Technologies, Barcelona, Spain). MassHunter Mass Profiler Professional Software (Agilent Technologies, Barcelona, Spain) and Metabolanalyst Software (Xia and Wishart, 2016; Chong and Xia, 2018) were used to perform a non-targeted lipidomic analysis of the obtained data. Only those features with a minimum of 2 ions were selected. After that, the molecular characteristics in the samples were aligned using a retention time window of 0.1 % ± 0.25 min and 30.0 ppm ± 2.0mDa. Only features found in at least 70% of the QC samples accounted for the correction of individual bias, and the signal was corrected using a LOESS approach [83, 84]. For annotation, relevant features, defined by exact mass and retention time, were searched against the HMDB [85] (accuracy < 30ppm) and LIPID MAPS [86] databases (accuracy < 20ppm). The identities obtained were compared to the authentic standards' retention times. Finally, identities were confirmed by searching experimental MS/MS spectra against *in silico* libraries, using HMDB and LipidMatch, an R-based tool for lipid identification [87].

### Immunohistochemistry

Formalin-fixed, paraffin-embedded, de-waxed sections 4 µm thick of the frontal cortex (GM) and subcortical WM in five MA cases were processed for specific immune-histochemistry. The sections were boiled in citrate buffer (20 min) to retrieve protein antigenicity. Endogenous peroxidases were blocked by incubation in 10% methanol-1% H_2_ O_2_ solution (15 min) followed by 3% normal horse serum solution. Then the sections were incubated at 4ºC overnight with one of the primary rabbit polyclonal antibodies: 3-ketoacyl-CoA thiolase (ACCA1) (MyBioSource MBS1492126) used at a dilution of 1/100; Fatty Acid Synthase (FAS) (C20G5, Cell Signaling 3180) diluted 1/50, and Stearoyl-CoA desaturase (SCD) (MyBioSource, BS421254) used at a dilution of 1/50. After incubation with the primary antibody, the sections were incubated with EnVision+ system peroxidase (Dako, Agilent Technologies, Santa Clara, CA, USA) for 30 min at room temperature. The peroxidase reaction was visualized with diaminobenzidine and H_2_ O_2_. Control of the immunostaining included omission of the primary antibody; no signal was obtained following incubation with only the secondary antibody. Sections were slightly counterstained with hematoxylin. Due to the individual variability of the immunostaining, no attempt at quantification was performed; immunohistochemistry was used to assess the localization of the enzymes.

### RNA extraction and RT-qPCR validation

RNA from frozen frontal cortex area 8 (GM) and subcortical WM was extracted following the supplier's instructions (RNeasy Mini Kit, Qiagen® GmbH, Hilden, Germany). RNA integrity and 28S/18S ratios were determined with the Agilent Bioanalyzer (Agilent Technologies Inc, Santa Clara, CA, USA) to assess RNA quality, and the RNA concentration was evaluated using a NanoDrop™ Spectrophotometer (Thermo Fisher Scientific). Complementary DNA (cDNA) preparation used a High-Capacity cDNA Reverse Transcription kit (Applied Biosystems, Foster City, CA, USA) following the protocol provided by the supplier. TaqMan RT-qPCR assays were duplicated for each gene on cDNA samples in 384-well optical plates using an ABI Prism 7900 Sequence Detection system (Applied Biosystems, Life Technologies, Waltham, MA, USA). For each 10μL TaqMan reaction, 2.25μL cDNA was mixed with 0.25μL 20x TaqMan Gene Expression Assays and 2.50μL of 2x TaqMan Universal PCR Master Mix (Applied Biosystems). The identification numbers and names of TaqMan probes are shown in Supplementary Table 2. Values of β-glucuronidase (*GUS-β* mRNA) were used as internal controls for normalization. The parameters of the reactions were 50°C for 2 min, 95°C for 10 min, and 40 cycles of 95°C for 15 sec and 60°C for 1 min. Finally, Sequence Detection Software (SDS version 2.2.2, Applied Biosystems) was used to capture TaqMan PCR data. The data were analyzed with the double-delta cycle threshold (ΔΔCT) method.

### Statistics

For lipidomics and fatty acids analysis, multivariate statistics (Principal Component Analysis (PCA), Partial Least-squares Discriminant Analysis (PLS-DA), and Hierarchical and Classification Analyses) were performed using Metaboanalyst software [88]. For comparing the lipidomic and fatty acyl composition between WM and GM, Mann-Whitney U tests were performed using only those samples from MA groups.

Regarding the changes according to progression, two approaches were used: First, a multi-group comparison between all the Braak Stages was performed taking into account all lipids/fatty acids. Kruskal-Wallis tests with Dunn’s post-hoc test was performed. Second, the correlation between lipids/fatty acids and Braak Stages was assessed. Spearman correlations were performed on all fatty acids, and only on those lipids/fatty acids deemed significant in the multi group comparison that were identified. R version 4.0.3 was used [89]. For transcriptomics, multi-group comparison between all the Braak Stages was performed using Kruskal-Wallis tests with Dunn’s post-hoc test.

Due to the small N per group (N<6), normality could not be determined, and non-parametric tests were used. Significance was set at p < 0.05, and Benjamini-Hochberg's false discovery rate (FDR) corrected p-values were also calculated and reported. Due to the large amount of statistically significant lipids in the comparison between WM and GM, only those with p-value<0.01 were annotated and reported.

## RESULTS

### Lipidomic profiles differ in grey matter (GM) and white matter (WM)

The first goal of the present study was to characterize the potential differences between the GM of the frontal cortex and WM of the frontal lobe’s *centrum semi-ovale* in healthy adult individuals according to their lipid composition. Firstly, we applied an untargeted lipidomic methodology to obtain global information about the differences between GM and WM lipidomes. Then, we characterized the fatty acid profiles of lipidomes from both GM and WM using a targeted approach.

**Figure 1.**
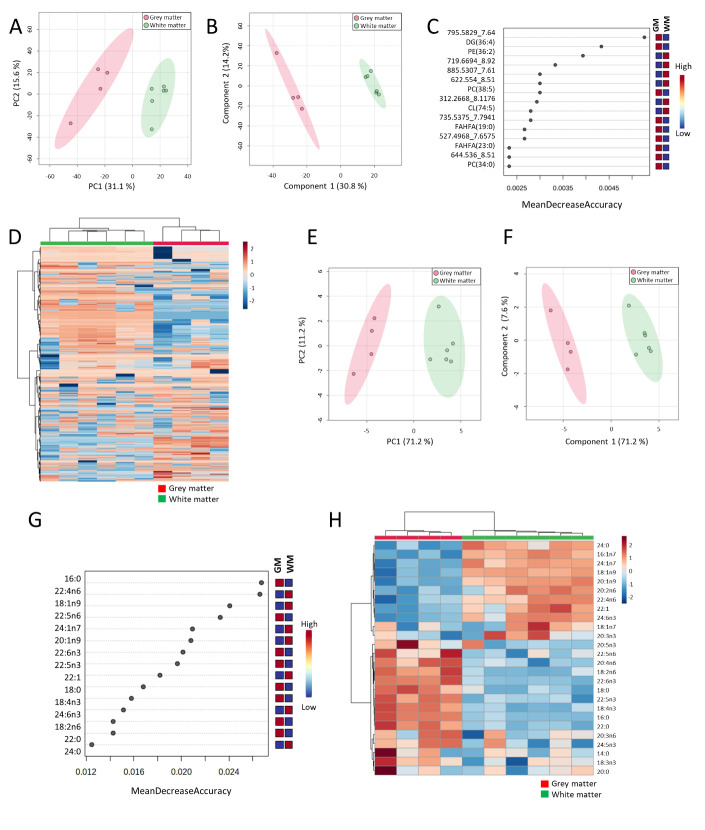
Untargeted and targeted lipid profiles distinguish grey and white matter brain tissues. (A) Principal component analysis (PCA) scores plot of samples whole lipidome. (B) Partial least squares discriminant analysis (PLS-DA) scores plot of samples lipidome. Leave One Out Cross-Validation (LOOCV) accuracy: 1.0, R2: 0.97, and Q2: 0.81 (one component). (C) Random Forest classification of samples VIP plot according to Mean Decrease in Accuracy using brain white and grey matter whole lipidome. OBB Error: 0.0. (D) Heatmap clustering analysis of samples whole lipidome. (E) Principal component analysis (PCA) scores plot of samples fatty acids (FA) profile. F) Partial least squares discriminant analysis (PLS-DA) scores plot of samples FA profile. Leave One Out Cross-Validation (LOOCV) accuracy: 1.0, R2: 0.96, and Q2: 0.93 (one component). (G) Random Forest classification of samples VIP plot according to Mean Decrease in Accuracy using brain white and grey matter FA profile. GM: grey matter, WM: white matter. OBB Error: 0.0. (H) Heatmap clustering analysis of samples fatty acids (FA) profile. n (GM) = 4, n (WM) = 6.

Baseline correction, peak picking, and peak alignment were performed on untargeted approach acquired data resulting in a total of 9,207 molecules from negative and positive ionization modes. After quality control assessment, filtering, and correcting the signal, 2,048 features were used for multivariate and univariate statistical analysis. Multivariate statistics (Fig. 1A-D) revealed marked differences in lipidome profiles, indicating that GM and WM show region-specific lipidomes. These results were reinforced by the detection, characterization, and quantification of 24 different fatty acids (Fig. 1E-H).

**Table 2:** Fatty acid composition, general indexes, and estimated enzyme activities in the cerebral cortex area 8 (grey matter: GM) and white matter of the *centrum semi-ovale* of the frontal lobe (WM) in middle-aged individuals without NFTs and SPs in any brain region.

	GM	WM	Mann-Whitney p-value	Mann-Whitney FDR p-value
14:0	2.51 [2.27;2.82]	2.26 [2.06;2.40]	0.394	0.402
16:0	26.0 [25.9;26.8]	14.9 [14.8;15.3]	0.011	0.015
16:1n-7	1.54 [1.46;1.64]	2.88 [2.75;3.02]	0.011	0.015
18:0	25.0 [24.6;25.2]	21.6 [21.3;22.2]	0.011	0.015
18:1n-9	20.0 [18.5;20.8]	32.6 [31.8;33.2]	0.011	0.015
18:1n-7	4.92 [4.62;5.19]	5.31 [4.78;5.57]	0.286	0.304
18:2n-6	0.96 [0.82;1.16]	0.26 [0.25;0.33]	0.011	0.015
18:3n-3	0.12 [0.11;0.13]	0.10 [0.08;0.11]	0.019	0.025
18:4n-3	1.48 [1.37;1.61]	0.43 [0.37;0.50]	0.011	0.015
20:0	0.29 [0.28;0.31]	0.28 [0.27;0.29]	0.522	0.522
20:1n-9	1.22 [1.12;1.27]	3.76 [3.28;3.83]	0.011	0.015
20:2n-6	0.48 [0.46;0.51]	1.03 [0.86;1.11]	0.019	0.025
20:3n-3	0.59 [0.56;0.62]	0.68 [0.62;0.80]	0.136	0.151
20:4n-6	4.54 [4.23;4.70]	2.90 [2.81;2.95]	0.011	0.015
20:3n-6	0.36 [0.30;0.40]	0.25 [0.21;0.28]	0.088	0.104
22:0	0.23 [0.20;0.27]	0.07 [0.07;0.08]	0.011	0.015
20:5n-3	0.87 [0.76;1.21]	0.48 [0.46;0.52]	0.055	0.067
22:1n-9	0.11 [0.10;0.12]	0.19 [0.18;0.22]	0.011	0.015
22:4n-6	2.20 [1.95;2.40]	3.29 [3.02;3.53]	0.011	0.015
22:5n-6	0.55 [0.40;0.73]	0.28 [0.26;0.33]	0.011	0.015
22:5n-3	0.28 [0.25;0.30]	0.09 [0.07;0.09]	0.011	0.015
24:0	0.40 [0.38;0.45]	1.01 [0.95;1.04]	0.011	0.015
22:6n-3	2.90 [2.79;3.18]	0.71 [0.69;0.73]	0.011	0.015
24:1n-9	0.75 [0.68;0.81]	3.08 [2.95;3.26]	0.011	0.015
24:5n-3	1.51 [1.22;1.75]	0.68 [0.47;0.87]	0.033	0.041
24:6n-3	0.14 [0.13;0.16]	0.50 [0.43;0.54]	0.011	0.015
SFA	54.7 [54.1;55.7]	40.4 [40.0;41.5]	0.011	0.015
UFA	45.3 [44.3;45.9]	59.6 [58.5;60.0]	0.011	0.015
PUFA	17.1 [16.8;17.5]	11.6 [11.5;12.1]	0.011	0.015
MUFA	28.6 [27.3;29.0]	47.8 [46.8;48.6]	0.011	0.015
PUFAn-3	8.33 [8.23;8.35]	3.77 [3.66;4.21]	0.011	0.015
PUFAn-6	9.01 [8.48;9.48]	7.85 [7.76;8.23]	0.136	0.151
ACL	17.8 [17.7;17.9]	18.1 [18.1;18.1]	0.011	0.015
DBI	101 [99.0;104]	95.1 [94.7;95.7]	0.011	0.015
PI (a)	80.9 [80.2;83.2]	50.5 [48.6;52.6]	0.011	0.015
PI (b)	56.9 [56.2;58.5]	36.5 [35.3;37.7]	0.011	0.015
Δ9(n-7)	0.06 [0.06;0.06]	0.19 [0.18;0.20]	0.011	0.015
Δ9(n-9)	0.80 [0.74;0.83]	1.49 [1.43;1.56]	0.011	0.015
Δ5(n-6)	12.6 [11.7;14.2]	10.3 [10.2;13.1]	0.286	0.304
Δ6(n-3) (a)	0.09 [0.09;0.10]	0.69 [0.62;1.03]	0.011	0.015
Δ6(n-3) (b)	12.0 [11.5;12.2]	5.13 [3.86;5.31]	0.011	0.015
Elovl3(n-9)	0.06 [0.06;0.06]	0.12 [0.10;0.12]	0.011	0.015
Elovl6	0.93 [0.90;0.96]	1.45 [1.43;1.48]	0.011	0.015
Elovl1-3-7a	0.01 [0.01;0.01]	0.01 [0.01;0.01]	0.394	0.402
Elovl1-3-7b	0.82 [0.74;0.89]	0.27 [0.26;0.27]	0.011	0.015
Elovl1-3-7c	1.88 [1.39;2.37]	14.2 [11.8;16.1]	0.011	0.015
Elovl5(n-6)	0.48 [0.42;0.57]	3.72 [2.65;4.35]	0.011	0.015
Elovl2-5 (n-6)	0.51 [0.48;0.52]	1.12 [1.01;1.25]	0.011	0.015
Elovl 2-5(n-3)	0.36 [0.30;0.38]	0.17 [0.12;0.20]	0.136	0.151
Elovl 2(n-3)	5.43 [4.63;6.12]	8.95 [8.34;9.39]	0.033	0.041
Perox β-ox	1.48 [1.33;1.60]	0.22 [0.20;0.23]	0.011	0.015

Values are median [Q1;Q3] from 4-6 different individuals and are expressed as mole percent; Mann-Whitney U test p-values <0.05 are highlighted in bold. NFTs: neurofibrillary tangle; SPs: senile plaquesACL: average chain length; SFA: saturated fatty acids; UFA: unsaturated fatty acids; MUFA: monounsaturated fatty acids; PUFA: polyunsaturated fatty acids; PUFAn-6: PUFA n-6 series; PUFAn-3: PUFA n-3 series; DBI: double-bond index; PI: peroxidizability index. Number of samples: GM = 4; WM = 6; FDR: False Discovery Rate. FDR was corrected for 51 tests.

**Table 3:** Fatty acid composition, general indexes, and estimated enzyme activities in the frontal cortex (grey matter: GM) in middle-aged individuals without NFTs and SPs (A) and in cases at AD stages I-II/0-A (B), III-IV/0-B (C), and V-VI/B-C (D).

Fatty acid	A	B	C	D	Kruskal-Wallis p-value	Kruskal-Wallis FDR p-value	Spearman’s Rho	Correlation p-value	Correlation FDR corrected p-value
	*n=4*	*n=6*	*n=6*	*n=6*					
14:0	2.51 [2.27;2.82]	2.14 [1.94;2.27]	2.44 [2.33;2.97]	2.63 [2.16;2.68]	0.363	0.514	0.135	0.547	0.716
16:0	26.0 [25.9;26.8]	25.1 [23.8;26.5]	28.4 [28.0;29.0]	27.3 [26.8;28.0]	0.039	0.328	0.348	0.112	0.249
16:1n-7	1.54 [1.46;1.64]	1.73 [1.40;1.95]	1.27 [1.23;1.35]	1.47 [1.31;1.52]	0.220	0.404	-0.254	0.252	0.444
18:0	25.0 [24.6;25.2]	24.5 [23.8;25.4]	25.3 [24.9;25.7]	24.3 [23.1;25.1]	0.415	0.572	-0.077	0.733	0.857
18:1n-9	20.0 [18.5;20.8]	21.5 [18.6;23.9]	16.8 [16.2;17.6]	17.0 [15.6;19.4]	0.16	0.397	-0.362	0.097	0.249
18:1n-7	4.92 [4.62;5.19]	5.13 [4.68;5.46]	4.48 [4.43;4.55]	5.10 [5.07;5.54]	0.065	0.337	0.098	0.664	0.806
18:2n-6	0.96 [0.82;1.16]	0.98 [0.75;1.19]	1.05 [0.91;1.10]	0.83 [0.74;1.07]	0.844	0.897	-0.134	0.551	0.716
18:3n-3	0.12 [0.11;0.13]	0.12 [0.12;0.13]	0.12 [0.11;0.13]	0.12 [0.11;0.13]	0.94	0.959	-0.109	0.626	0.779
18:4n-3	1.48 [1.37;1.61]	1.36 [1.29;1.43]	1.57 [1.48;1.59]	1.68 [1.39;1.72]	0.298	0.46	0.325	0.138	0.295
20:0	0.29 [0.28;0.31]	0.30 [0.29;0.30]	0.29 [0.27;0.32]	0.29 [0.26;0.32]	0.883	0.919	-0.066	0.768	0.857
20:1n-9	1.22 [1.12;1.27]	1.13 [0.99;1.44]	0.82 [0.69;0.98]	0.83 [0.68;1.01]	0.205	0.404	-0.415	0.054	0.213
20:2n-6	0.48 [0.46;0.51]	0.46 [0.40;0.53]	0.33 [0.30;0.39]	0.41 [0.34;0.53]	0.066	0.337	-0.301	0.172	0.346
20:3n-3	0.59 [0.56;0.62]	0.66 [0.61;0.76]	0.63 [0.57;0.66]	0.62 [0.59;0.68]	0.565	0.686	0.047	0.832	0.8573
20:4n-6	4.54 [4.23;4.70]	4.56 [4.41;4.68]	5.25 [5.09;5.54]	4.94 [4.73;5.13]	0.045	0.328	0.439	0.040	0.213
20:3n-6	0.36 [0.30;0.40]	0.27 [0.25;0.40]	0.27 [0.21;0.36]	0.26 [0.19;0.27]	0.654	0.776	-0.274	0.216	0.408
22:0	0.23 [0.20;0.27]	0.24 [0.21;0.25]	0.24 [0.23;0.29]	0.25 [0.20;0.30]	0.975	0.975	0.063	0.780	0.8573
20:5n-3	0.87 [0.76;1.21]	0.70 [0.67;0.74]	0.87 [0.80;0.97]	0.74 [0.70;0.89]	0.316	0.46	0.059	0.792	0.8573
22:1n-9	0.11 [0.10;0.12]	0.10 [0.09;0.11]	0.09 [0.08;0.09]	0.11 [0.10;0.12]	0.204	0.404	-0.134	0.551	0.716
22:4n-6	2.20 [1.95;2.40]	2.18 [2.10;2.48]	2.05 [1.91;2.33]	1.96 [1.85;2.10]	0.531	0.677	-0.231	0.300	0.510
22:5n-6	0.55 [0.40;0.73]	0.43 [0.36;0.48]	0.54 [0.43;0.76]	0.54 [0.50;0.62]	0.168	0.397	0.269	0.224	0.409
22:5n-3	0.28 [0.25;0.30]	0.27 [0.23;0.30]	0.16 [0.13;0.28]	0.12 [0.12;0.13]	0.01	0.255	-0.693	0.0003	0.017
24:0	0.40 [0.38;0.45]	0.45 [0.43;0.50]	0.38 [0.37;0.38]	0.37 [0.36;0.42]	0.17	0.397	-0.383	0.078	0.235
22:6n-3	2.90 [2.79;3.18]	3.47 [2.29;3.73]	4.24 [3.97;4.36]	3.77 [3.56;4.08]	0.053	0.337	0.450	0.035	0.213
24:1n-9	0.75 [0.68;0.81]	0.96 [0.62;1.22]	0.40 [0.30;0.42]	0.42 [0.31;0.59]	0.045	0.328	-0.481	0.023	0.198
24:5n-3	1.51 [1.22;1.75]	0.83 [0.81;1.39]	1.28 [0.88;1.50]	1.27 [1.01;1.47]	0.709	0.799	-0.063	0.780	0.857
24:6n-3	0.14 [0.13;0.16]	0.14 [0.13;0.21]	0.10 [0.09;0.11]	0.14 [0.13;0.17]	0.097	0.353	-0.130	0.561	0.716
SFA	54.7 [54.1;55.7]	52.9 [50.4;55.1]	57.1 [56.2;58.5]	54.9 [53.5;56.5]	0.097	0.353	0.177	0.429	0.657
UFA	45.3 [44.3;45.9]	47.1 [44.9;49.6]	42.9 [41.5;43.8]	45.1 [43.5;46.5]	0.097	0.353	-0.177	0.429	0.657
PUFA	17.1 [16.8;17.5]	16.3 [15.5;18.0]	18.5 [18.3;18.8]	18.3 [17.6;19.1]	0.172	0.397	0.373	0.086	0.245
MUFA	28.6 [27.3;29.0]	30.8 [26.9;34.0]	24.0 [22.9;25.0]	25.2 [23.2;27.6]	0.12	0.381	-0.357	0.102	0.249
PUFAn3	8.33 [8.23;8.35]	7.47 [6.35;9.25]	8.95 [8.78;9.30]	9.10 [8.12;9.93]	0.315	0.46	0.355	0.104	0.249
PUFAn6	9.01 [8.48;9.48]	9.04 [8.61;9.72]	9.42 [9.12;10.3]	9.29 [8.64;9.97]	0.545	0.678	0.143	0.523	0.716
ACL	17.8 [17.7;17.9]	17.9 [17.9;17.9]	17.8 [17.7;17.9]	17.9 [17.8;17.9]	0.432	0.58	0.023	0.917	0.917
DBI	101 [99.0;104]	101 [99.2;106]	106 [105;108]	105 [104;109]	0.215	0.404	0.425	0.048	0.213
PI(a)	80.9 [80.2;83.2]	78.0 [70.6;88.8]	92.0 [89.8;93.7]	90.2 [85.2;95.1]	0.179	0.397	0.397	0.067	0.220
PI(b)	56.9 [56.2;58.5]	54.6 [50.1;61.7]	63.8 [62.4;65.3]	63.0 [59.5;66.1]	0.143	0.397	0.419	0.052	0.213
Δ9(n-7)	0.06 [0.06;0.06]	0.07 [0.05;0.08]	0.04 [0.04;0.05]	0.05 [0.05;0.06]	0.104	0.354	-0.349	0.111	0.249
Δ9(n-9)	0.80 [0.74;0.83]	0.87 [0.74;1.01]	0.67 [0.62;0.71]	0.69 [0.65;0.79]	0.238	0.419	-0.298	0.176	0.346
Δ5(n-6)	12.6 [11.7;14.2]	16.9 [11.9;18.0]	20.4 [14.4;25.3]	19.1 [17.7;23.7]	0.275	0.438	0.406	0.060	0.220
Δ6(n-3)	12.0 [11.5;12.2]	11.3 [11.1;11.4]	13.8 [11.2;14.6]	13.5 [12.1;15.2]	0.082	0.353	0.456	0.032	0.213
Δ6(n-3)	0.09 [0.09;0.10]	0.16 [0.11;0.25]	0.07 [0.07;0.10]	0.09 [0.09;0.15]	0.463	0.605	-0.145	0.516	0.716
Elovl3(n-9)	0.06 [0.06;0.06]	0.05 [0.05;0.06]	0.05 [0.05;0.06]	0.05 [0.04;0.05]	0.247	0.42	-0.394	0.069	0.220
Elovl6	0.93 [0.90;0.96]	0.99 [0.95;0.99]	0.88 [0.88;0.90]	0.89 [0.86;0.91]	0.006	0.255	-0.587	0.004	0.103
Elovl1-3-7a	0.01 [0.01;0.01]	0.01 [0.01;0.01]	0.01 [0.01;0.01]	0.01 [0.01;0.01]	0.68	0.788	-0.045	0.840	0.8573
Elovl1-3-7b	0.82 [0.74;0.89]	0.78 [0.67;0.85]	0.87 [0.75;0.91]	0.86 [0.81;0.99]	0.769	0.834	0.174	0.438	0.657
Elovl1-3-7c	1.88 [1.39;2.37]	2.11 [1.66;2.23]	1.47 [1.27;1.59]	1.57 [1.23;2.08]	0.721	0.799	-0.198	0.375	0.618
Elovl5(n-6)	0.48 [0.42;0.57]	0.43 [0.33;0.71]	0.34 [0.32;0.39]	0.49 [0.42;0.62]	0.274	0.438	-0.052	0.816	0.857
Elovl2-5 (n-6)	0.51 [0.48;0.52]	0.47 [0.40;0.56]	0.40 [0.38;0.42]	0.39 [0.33;0.47]	0.222	0.404	-0.426	0.047	0.213
Elovl 2-5(n-3)	0.36 [0.30;0.38]	0.40 [0.32;0.43]	0.26 [0.17;0.30]	0.16 [0.14;0.18]	0.036	0.328	-0.519	0.013	0.167
Elovl 2(n-3)	5.43 [4.63;6.12]	3.97 [3.30;4.44]	6.78 [5.13;10.8]	9.72 [8.25;11.9]	0.043	0.328	0.537	0.009	0.167
Perox β-Ox	1.48 [1.33;1.60]	1.64 [0.97;1.70]	2.00 [1.73;2.12]	2.07 [1.76;2.16]	0.127	0.381	0.495	0.019	0.195

ACL: average chain lenght; SFA: saturated fatty acids; UFA: unsaturated fatty acids; MUFA: monounsaturated fatty acids; PUFA: polyunsaturated fatty acids; PUFAn-6: PUFA n-6 series; PUFAn-3: PUFA n-3 series; DBI: double-bond index; PI: peroxidizability index; NFTs: neurofibrillary tangle SPs: senile plaques; FDR: False Discovery Rate. FDR was corrected for 51 tests.

**Figure 2.**
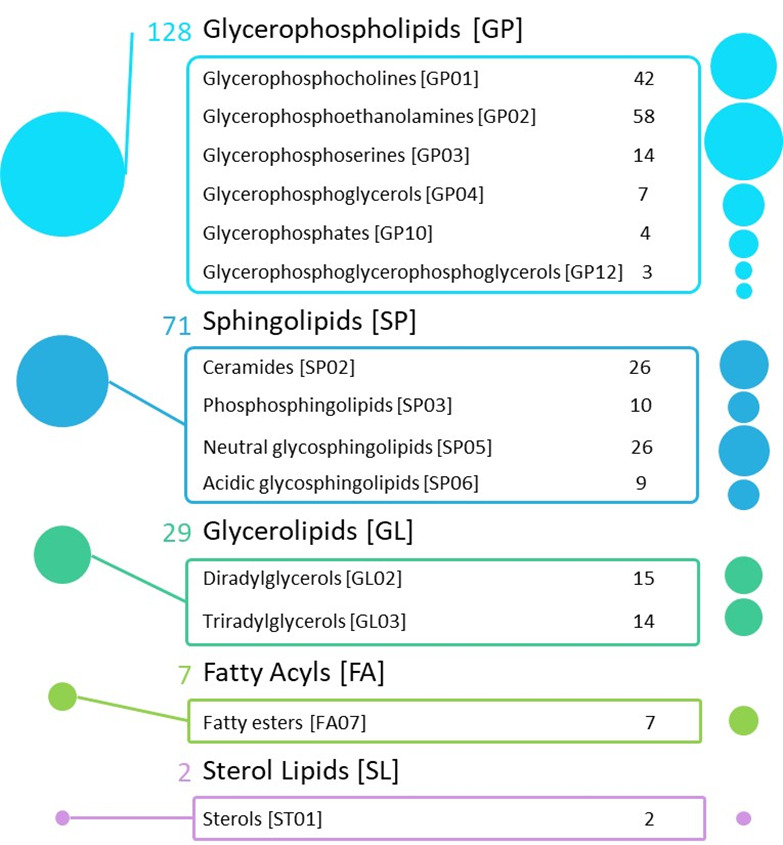
Infographic with the differential lipids between white matter (WM) and grey matter (GM). The 237 identified lipid features were named and organized into five major categories, according to the LIPIDMAPS database. The circles show the area proportional to the number of compounds identified by each category (left) and class (right). A different color has been assigned to each category.

Univariate statistics of untargeted lipidomics revealed 652 statistically different lipid species between GM and WM. Annotation was performed on those 270 lipids with p-value<0.01. Among them, 237 lipids were successfully identified (based on exact mass, retention time, isotopic distribution, and MS/MS spectrum) (Supplementary Table 3). Identified lipids belong to five major categories (Fig. 2): i) Fatty acyls, covering seven esters of fatty acids, as fatty acid esters of hydroxyl fatty acids (FAHFA), and one AcylCoAs, the Retinoyl-CoA; ii) GLs, including 15 diacylglycerols (DG) and 14 triacylglycerols (TG); iii) Glycerophospholipids (GP), including 42 PCs, 58 glycerophosphoethanolamines (PEs), 7 glycerophosphoglycerols (PGs), 3 cardiolipins (CLs), 14 PS, and 4 phosphatidic acids (PAs); iv) Sphingolipids, counting 25 Cer, 1 ceramide phosphate (CerP), 26 glycosylceramides (HexCer), 9 sulfatides, and 10 SM; and v) Sterol lipids: 2 cholesteryl esters (CEs). The remaining 33 were unknown lipid species (Supplementary Table 4). The WM, in comparison with GM, exhibited lower concentration of DG, PA, and CE, and significant enrichment in TG(O), PC, PE, sulfatides, Cer, glycosphingolipids, and SM (Supplementary Table 3). Remarkably, several lipid species showing enrichment in WM corresponded to alkyl and alkenyl ethers, mostly presented as TG, PC, and PE species. For FAHFA, TG, PG, CL, and PS, the lower or enriched condition in WM is lipid species-dependent (Supplementary Table 3).

The fatty acid profile showed marked differences between WM and GM (Table 2). Lower levels of 16:0 (42%), 18:0 (13%), 18:2n-6 (73%), 18:3n-3 (17%), 18:4n-3 (71%), 20:4n-6 (16%), 22:0 (70%), 22:5n-6 (49%), 22:5n-3 (68%), 22:6n-3 (75%) and 24:5n-3 (55%) whereas higher levels of 16:1n-7 (87%), 18:1n-9 (63%), 20:1n-9 (200%), 20:2n-6 (115%), 22:1n-9 (73%), 22:4n-6 (49%), 24:0 (152%), 24:1n-9 (319%), and 24:6n-3 (257%) were detected in the WM compared with the GM. Differences in fatty acid composition results in lower levels of SFA (26%) and PUFA (31%), including PUFAn-3 (55%) and PUFAn-6 (13%), and higher levels of MUFA (67%), and ACL (1%) in WM, leading to lower values of DBI (6%) and PI (36%).

**Figure 3.**
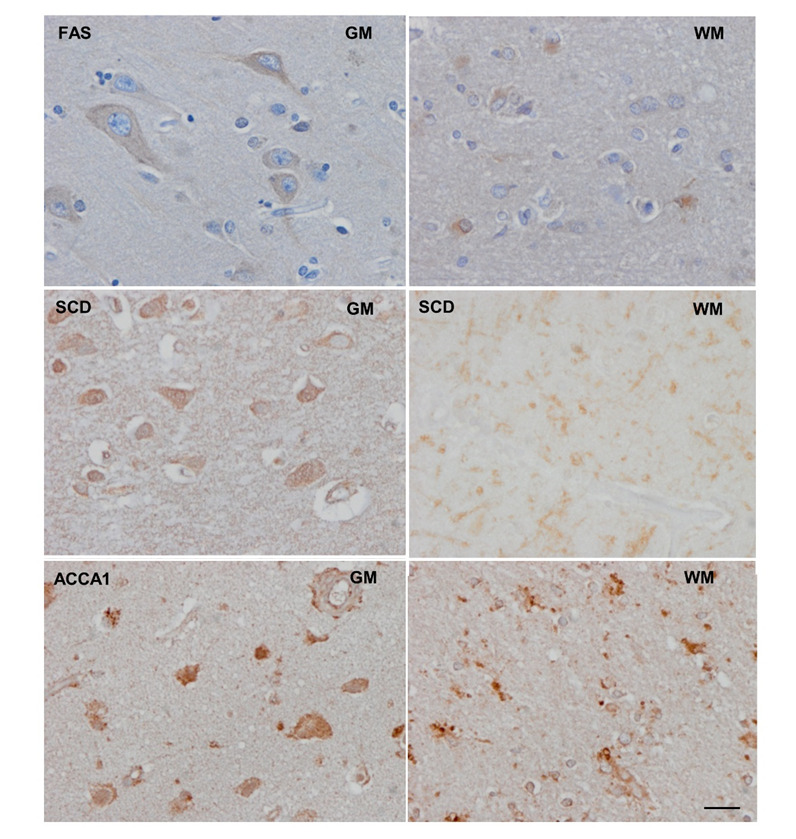
Representative sections of the grey matter (GM) and white matter (WM) in a control subject. ACAA1 (3-ketoacyl-CoA thiolase) immunoreactivity was found in neurons and glial cells. In neurons, moderate immunoreactivity decorated the cytoplasm; in glial cells, ACAA1 immunoreactivity formed small cytoplasmic granules. FAS (fatty acid synthase) immunoreactivity was found in the cytoplasm of neurons and astrocytes; SCD (stearoyl-CoA desaturase) in neurons; no immunostaining was detected in the WM. In these figures, neurons were recognized by their large cytoplasm, large and clear nucleus and apparent nucleolus; astrocytes were recognized by their small size, small nucleus, and radiated cytoplasm. Paraffin sections slightly counterstained with haematoxylin, bar = 25μm.

Modifications in desaturase and elongase activities could be behind these specific lipotypes. Thus, the estimation of these activities showed that the WM was characterized by a lower desaturase delta-6(n-3), elongase 1-3-7b, and peroxisomal β-oxidation activities, whereas desaturase delta-9 and the rest of the elongases activity were significantly higher compared with the GM. Importantly, immunohistochemistry experiments performed in three specifically selected enzymes (the peroxisomal β-oxidation related enzyme ACAA1, the FAS and the desaturase delta-9 or SCD) reinforced these results: ACAA1 immunoreactivity was found in neurons and glial cells. In neurons, moderate immunoreactivity decorated the cytoplasm; in glial cells, ACAA1 immunoreactivity formed small cytoplasmic granules. FAS immunoreactivity was found in the cytoplasm of neurons and astrocytes; whereas SCD immunoreactivity was only found in GM (Fig. 3). Thus, ACAA1, FAS, and SCD are localized in neurons and glial cells, but the immunoreactivity is higher in GM suggesting an increased content and activity of these metabolic pathways, in line with the analytical results obtained from fatty acid profile.

**Table 4:** Fatty acid composition, general indexes, and estimated enzyme activities in the white matter (WM) in middle-aged individuals without NFTs and SPs (A) and in cases at AD stages I-II/0-A (B), III-IV/0-B (C), and V-VI/B-C (D).

Fatty acid	A	B	C	D	Kruskal-Wallis p-value	Kruskal-Wallis FDR p-value	Spearman’s Rho	Correlation p-value	Correlation corrected p-value
	*N=6*	*N=7*	*N=6*	*N=6*					
14:0	2.26 [2.06;2.40]	2.16 [1.93;2.36]	1.89 [1.46;2.08]	2.21 [2.06;2.33]	0.175	0.425	-0.144	0.491	0.783
16:0	14.9 [14.8;15.3]	15.4 [15.2;15.6]	16.4 [16.2;16.5]	15.6 [15.0;16.3]	0.194	0.43	0.293	0.153	0.482
16:1n-7	2.88 [2.75;3.02]	3.04 [2.84;3.13]	3.02 [2.96;3.07]	3.16 [3.11;3.31]	0.159	0.425	0.405	0.044	0.331
18:0	21.6 [21.3;22.2]	21.3 [21.2;21.4]	21.8 [21.2;22.0]	21.5 [21.2;21.7]	0.594	0.779	-0.110	0.598	0.811
18:1n-9	32.6 [31.8;33.2]	32.8 [32.4;33.6]	32.9 [32.5;33.0]	32.2 [31.9;32.4]	0.253	0.496	-0.159	0.447	0.735
18:1n-7	5.31 [4.78;5.57]	5.36 [4.96;5.64]	5.50 [5.43;5.77]	5.86 [5.63;6.37]	0.163	0.425	0.430	0.031	0.321
18:2n-6	0.26 [0.25;0.33]	0.28 [0.24;0.42]	0.38 [0.35;0.43]	0.25 [0.24;0.30]	0.236	0.481	0.048	0.819	0.889
18:3n-3	0.10 [0.08;0.11]	0.12 [0.11;0.14]	0.13 [0.11;0.14]	0.11 [0.11;0.12]	0.076	0.425	0.283	0.168	0.482
18:4n-3	0.43 [0.37;0.50]	0.44 [0.43;0.49]	0.47 [0.41;0.59]	0.51 [0.49;0.54]	0.328	0.532	0.366	0.071	0.331
20:0	0.28 [0.27;0.29]	0.28 [0.26;0.29]	0.26 [0.24;0.27]	0.29 [0.28;0.31]	0.173	0.425	0.087	0.677	0.825
20:1n-9	3.76 [3.28;3.83]	3.79 [2.83;4.15]	3.46 [3.15;3.50]	3.36 [2.94;3.61]	0.644	0.782	-0.262	0.205	0.524
20:2n-6	1.03 [0.86;1.11]	0.92 [0.88;0.94]	0.89 [0.89;0.96]	1.07 [0.97;1.21]	0.129	0.425	0.188	0.367	0.646
20:3n-3	0.68 [0.62;0.80]	0.63 [0.61;0.68]	0.55 [0.54;0.68]	0.61 [0.55;0.70]	0.473	0.67	-0.250	0.226	0.541
20:4n-6	2.90 [2.81;2.95]	2.76 [2.65;2.90]	2.95 [2.87;3.05]	2.81 [2.51;2.89]	0.334	0.532	-0.050	0.809	0.889
20:3n-6	0.25 [0.21;0.28]	0.20 [0.20;0.34]	0.22 [0.19;0.26]	0.21 [0.19;0.22]	0.602	0.779	-0.277	0.179	0.482
22:0	0.07 [0.07;0.08]	0.08 [0.07;0.09]	0.11 [0.08;0.11]	0.11 [0.10;0.11]	0.037	0.349	0.591	0.001	0.036
20:5n-3	0.48 [0.46;0.52]	0.41 [0.40;0.44]	0.45 [0.42;0.49]	0.51 [0.45;0.58]	0.367	0.567	0.060	0.774	0.877
22:1n-9	0.19 [0.18;0.22]	0.18 [0.17;0.19]	0.17 [0.16;0.18]	0.21 [0.18;0.23]	0.113	0.425	0.040	0.846	0.898
22:4n-6	3.29 [3.02;3.53]	2.97 [2.64;3.02]	2.88 [2.72;3.00]	2.76 [2.59;3.04]	0.108	0.425	-0.431	0.031	0.321
22:5n-6	0.28 [0.26;0.33]	0.19 [0.17;0.24]	0.23 [0.17;0.25]	0.22 [0.21;0.24]	0.089	0.425	-0.308	0.133	0.455
22:5n-3	0.09 [0.07;0.09]	0.10 [0.07;0.11]	0.11 [0.09;0.12]	0.09 [0.08;0.11]	0.434	0.651	0.236	0.254	0.541
24:0	1.01 [0.95;1.04]	0.85 [0.81;0.97]	0.76 [0.72;0.80]	0.90 [0.85;0.96]	0.041	0.349	-0.217	0.296	0.584
22:6n-3	0.71 [0.69;0.73]	0.77 [0.66;0.79]	0.89 [0.86;0.95]	0.77 [0.65;0.82]	0.015	0.293	0.192	0.357	0.646
24:1n-9	3.08 [2.95;3.26]	2.76 [2.57;3.01]	2.75 [2.46;3.09]	2.88 [2.75;3.73]	0.503	0.693	-0.035	0.865	0.900
24:5n-3	0.68 [0.47;0.87]	0.56 [0.52;1.32]	0.64 [0.51;1.01]	0.54 [0.51;0.58]	0.819	0.873	-0.159	0.447	0.735
24:6n-3	0.50 [0.43;0.54]	0.51 [0.39;0.54]	0.46 [0.40;0.54]	0.45 [0.44;0.57]	0.951	0.951	0.073	0.725	0.841
SFA	40.4 [40.0;41.5]	40.0 [39.9;40.8]	40.8 [40.5;41.0]	40.3 [39.8;41.6]	0.921	0.939	0.082	0.696	0.825
UFA	59.6 [58.5;60.0]	60.0 [59.2;60.1]	59.2 [59.0;59.5]	59.7 [58.4;60.2]	0.921	0.939	-0.082	0.696	0.825
PUFA	11.6 [11.5;12.1]	11.2 [11.0;12.2]	11.7 [11.4;11.9]	11.0 [10.6;11.2]	0.138	0.425	-0.333	0.103	0.400
MUFA	47.8 [46.8;48.6]	47.2 [46.8;48.9]	47.6 [47.3;48.1]	48.7 [47.3;49.5]	0.724	0.819	0.138	0.510	0.788
PUFAn-3	3.77 [3.66;4.21]	3.77 [3.38;4.34]	3.99 [3.76;4.18]	3.60 [3.58;4.02]	0.626	0.779	-0.106	0.611	0.811
PUFAn-6	7.85 [7.76;8.23]	7.29 [7.02;7.86]	7.55 [7.38;7.73]	7.37 [7.00;7.84]	0.457	0.666	-0.216	0.297	0.584
ACL	18.1 [18.1;18.1]	18.1 [18.0;18.2]	18.1 [18.0;18.2]	18.1 [18.0;18.1]	0.822	0.873	-0.104	0.620	0.811
DBI	95.1 [94.7;95.7]	93.5 [92.0;96.5]	94.7 [93.9;95.8]	92.3 [91.3;93.8]	0.102	0.425	-0.369	0.068	0.331
PI(a)	50.5 [48.6;52.6]	48.5 [46.8;52.4]	51.2 [49.4;51.6]	47.1 [44.7;47.9]	0.131	0.425	-0.327	0.109	0.400
PI(b)	36.5 [35.3;37.7]	34.9 [33.9;37.8]	36.8 [35.5;37.2]	34.0 [32.4;34.5]	0.135	0.425	-0.358	0.078	0.331
Δ9(n-7)	0.19 [0.18;0.20]	0.20 [0.18;0.20]	0.18 [0.18;0.19]	0.21 [0.19;0.21]	0.312	0.53	0.104	0.620	0.811
Δ9(n-9)	1.49 [1.43;1.56]	1.54 [1.49;1.59]	1.50 [1.48;1.55]	1.51 [1.50;1.52]	0.739	0.819	-0.022	0.915	0.934
Δ5(n-6)	10.3 [10.2;13.1]	13.2 [8.39;14.4]	13.5 [11.8;15.8]	13.9 [12.6;14.4]	0.614	0.779	0.239	0.248	0.541
Δ6(n-3)	5.13 [3.86;5.31]	3.61 [3.39;4.12]	3.65 [3.55;4.20]	4.49 [4.32;4.67]	0.186	0.43	0.002	0.989	0.989
Δ6(n-3)	0.69 [0.62;1.03]	0.65 [0.44;1.11]	0.68 [0.43;1.13]	0.94 [0.83;1.10]	0.714	0.819	0.189	0.365	0.646
Elovl3(n-9)	0.12 [0.10;0.12]	0.12 [0.09;0.12]	0.11 [0.10;0.11]	0.10 [0.09;0.11]	0.704	0.819	-0.237	0.252	0.541
Elovl6	1.45 [1.43;1.48]	1.39 [1.35;1.42]	1.32 [1.30;1.36]	1.38 [1.33;1.41]	0.146	0.425	-0.363	0.073	0.331
Elovl1-3-7a	0.01 [0.01;0.01]	0.01 [0.01;0.01]	0.01 [0.01;0.01]	0.01 [0.01;0.01]	0.097	0.425	0.132	0.528	0.792
Elovl1-3-7b	0.27 [0.26;0.27]	0.29 [0.28;0.31]	0.39 [0.35;0.43]	0.34 [0.31;0.38]	0.019	0.293	0.590	0.001	0.036
Elovl1-3-7c	14.2 [11.8;16.1]	11.4 [10.2;11.7]	8.07 [6.10;9.05]	8.77 [8.20;9.78]	0.022	0.293	-0.584	0.002	0.036
Elovl5(n-6)	3.72 [2.65;4.35]	3.20 [2.07;3.95]	2.48 [2.14;2.54]	4.51 [3.26;4.95]	0.206	0.438	0.082	0.694	0.825
Elovl2-5 (n-6)	1.12 [1.01;1.25]	1.08 [0.92;1.08]	1.00 [0.92;1.02]	1.00 [0.91;1.10]	0.279	0.508	-0.282	0.170	0.482
Elovl 2-5(n-3)	0.17 [0.12;0.20]	0.19 [0.18;0.22]	0.24 [0.21;0.27]	0.17 [0.14;0.23]	0.305	0.53	0.123	0.557	0.811
Elovl 2(n-3)	8.95 [8.34;9.39]	5.97 [5.33;20.0]	7.06 [6.16;8.30]	5.98 [5.06;6.91]	0.276	0.508	-0.387	0.055	0.331
Perox β-Ox	0.22 [0.20;0.23]	0.27 [0.23;0.28]	0.33 [0.29;0.35]	0.25 [0.23;0.28]	0.023	0.293	0.402	0.045	0.331

n: number of cases. Values are median [Q1;Q3] from 4-6 different individuals and are expressed as mole percent; p-values <0.05 are highlighted in bold. ACL: average chain length; SFA: saturated fatty acids; UFA: unsaturated fatty acids; MUFA: monounsaturated fatty acids; PUFA: polyunsaturated fatty acids; PUFAn-6: PUFA n-6 series; PUFAn-3: PUFA n-3 series; DBI: double-bond index; PI: peroxidizability index; NFTs: neurofibrillary tangle; SPs: senile plaques; FDR: False Discovery Rate. FDR was corrected for 51 tests.

### Changes in WM and GM lipidomes associated with sAD progression

We compared the lipidome profile of GM and WM from individuals without NFT and senile plaques (SP) pathology and AD cases grouped by their Braak stage: I-II, III-IV, and V-VI. Our results showed that disease progression had little impact in the differences of FA profile in GM and in WM. In the GM, sAD progression affected 20:4n-6 (Spearman’s Rho=0.439, correlation p-value=0.040), 22:5n-3 (Spearman’s Rho= -0.693, correlation p-value=0.0003), 22:6n-3 (Spearman’s Rho=0.450, correlation p-value=0.035) and 24:1n-9 (Spearman’s Rho=-0.481, correlation p-value=0.023) (Table 3). Interestingly, AD progression was also associated with an increase in GM’s DBI (Spearman’s Rho=0.425, correlation p-value=0.048), together with changes in delta-6 desaturase (Spearman’s Rho=0.456, correlation p-value=0.032), elovl6 (Spearman’s Rho= -0.587, correlation p-value=0.004), elovl2-5(n-6 and n-3) (Spearman’s Rho= -0.426, correlation p-value=0.047 and Spearman’s Rho= -0.519, correlation p-value=0.013), respectively), elovl2 (Spearman’s Rho=0.537, correlation p-value=0.009) estimation activities, and peroxisomal β-oxidation (Spearman’s Rho=0.495, correlation p-value=0.019) (Table 3). It is important to remark that, although not significant, there is a clear tendency of PI(b) (Spearman’s Rho=0.419, correlation p-value=0.052) to increase in GM with AD progression. In contrast, reinforcing lipid resilience of WM, AD progression was accompanied by differences only in 16:1n-7 (Spearman’s Rho=0.405, correlation p-value=0.044), 18:1n-9 (Spearman’s Rho=0.430, correlation p-value=0.031), 22:0 (Spearman’s Rho=0.591, correlation p-value=0.001) and 22:4n-6 (Spearman’s Rho= -0.431, correlation p-value=0.031) (Table 4). Behenic acid, 22:0, was the only molecule that positively correlated with AD progression. AD progression was also associated with changes in enzymatic activities involved in fatty acid biosynthesis specifically compromising elovl1-3-7b (Spearman’s Rho=0.590, correlation p-value=0.001), elovl1-3-7c (Spearman’s Rho= -0.584, correlation p-value=0.002), and peroxisomal β-oxidation (Spearman’s Rho=0.402, correlation p-value=0.045) (Table 4). After applying the FDR correction, elovl1-3-7b and elovl1-3-7c maintained a significant correlation with AD progression. Reinforcing the importance of anatomic location, note that the changes in fatty acid composition in GM in AD progression implicate changes that make the lipids more susceptible to lipoxidation. In contrast, WM seems to be protected and preserved.

**Figure 4.**
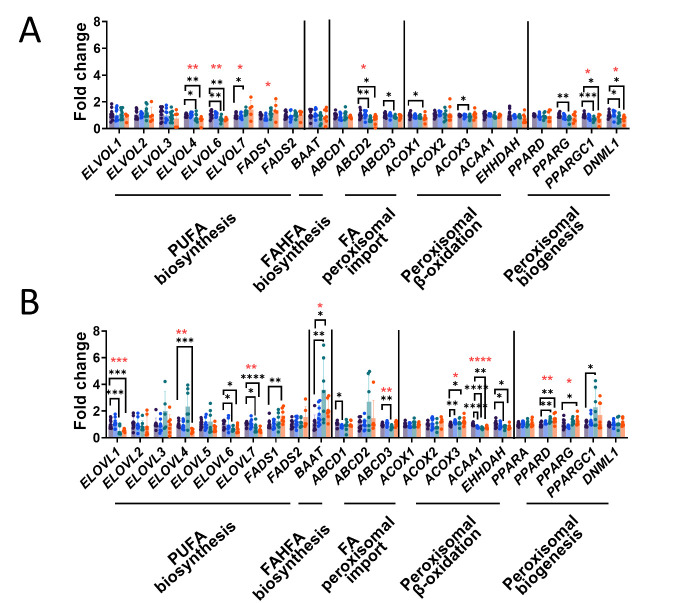
mRNA expression levels of PUFA biosynthesis, FAHFA biosynthesis and peroxisome-related genes in the (A) frontal cortex area 8 (GM), and (B) white matter of the frontal lobe (WM) in middle-aged (MA) individuals without NFTs and SPs in any brain region, and at Braak stages of I-II/0-A; III-IV/0-B, and V-VI/B-C. PUFA biosynthesis involved genes: elongation of very long-chain fatty acid (ELVOL) 1, 2, 3, 4, 5, 6, 7 and fatty acyl desaturases (FADS) 1 and 2. FAHFA biosynthesis related gene: bile acid CoA: amino acid N-acyltransferase (BAAT). Peroxisomal import of fatty acids (FA) involved genes: ATP-binding cassette transporters of subfamily D (ABCD) 1, 2 and 3. Peroxisomal β-oxidation related genes: Acyl-CoA oxidase (ACOX) 1, 2 and 3, and peroxisomal L-bifunctional enzyme (EHHADH). Peroxisomal biogenesis related genes: peroxisome proliferator-activated receptors (PPAR) D, G and GC1 and DNM1L. Kruskal-Wallis tests were applied. Red *: global statistical significance by Kruskal-Wallis; black *: significant after post-hoc Dunn’s test for the comparisons of the different Braak stages against the MA group (*p<0.05; **p<0.01;***p<0.001; ****p<0.0001). Bars are ordered from left to right as following: MA, ADI-II/0A, AD III-IV/0-B and AD V-VI/B-C.

**Figure 5.**
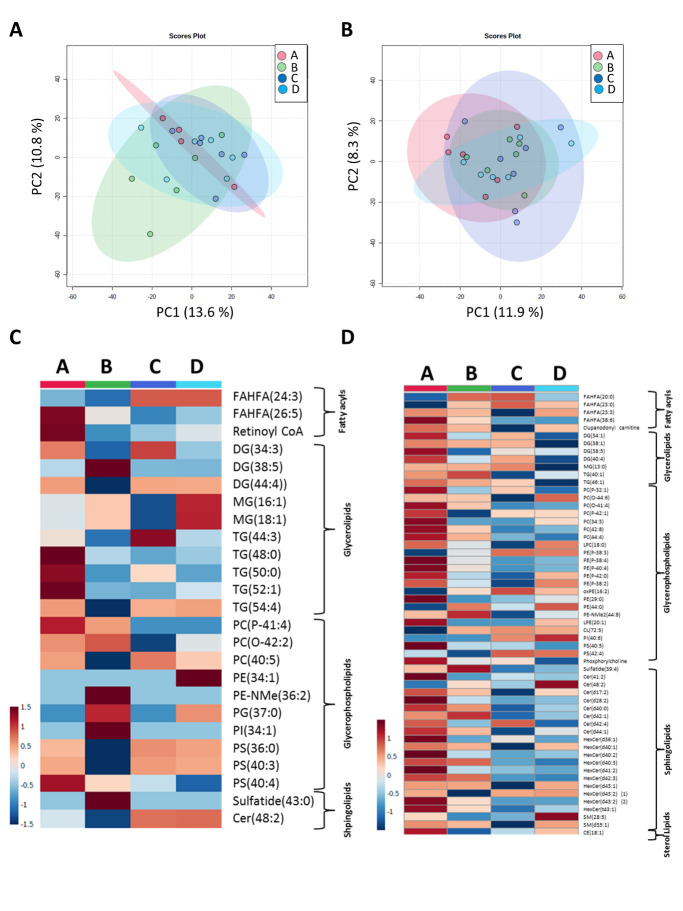
White matter (WM) is more affected by AD progression than grey matter (GM). Principal component analyses revealed no changes associated with AD progression in grey when whole lipidome is analyzed in both grey (A) and white (B) matters. Hierarchical clustering shows the relative abundance of significantly different lipid species in grey (C) and white (D) matter. A-D in panels C and D: A: Middle-aged individuals (MA) without NFTs and SPs; B: ADI-II/0-A; C: ADIII-IV/0-B; and D: ADV-VI/B-C. MA (n=6 for GM and n=4 for WM), ADI-II (n = 7); ADIII- IV (n = 5), and ADV-VI (n = 6)

To further elucidate the role of elongases and desaturases in AD progression, the mRNA levels of 7 elongation of very long-chain fatty acid (ELVOL) proteins and 2 fatty acyl desaturases (FADS), all related to PUFA biosynthesis, were analyzed (Fig. 4). Importantly, these analyses showed different modulation of mRNA expression in GM and WM in AD progression. Specifically, in GM we found decreased expression of two elongases (ELVOL4 (Kruskal-Wallis p=0.008, post-hoc Dunn’s test ADIII-IV vs MA p=0.04, ADV-VI vs MA p=0.006) and ELVOL6 (Kruskal-Wallis p=0.001, post-hoc Dunn’s test ADIII-IV vs MA p=0.005, ADV-VI vs MA p=0.08)) in middle-latter stages of AD and increased expression of the elongase ELVOL7 (Kruskal-Wallis p=0.02, post-hoc Dunn’s test ADIII-IV vs MA p=0.02) and the desaturase FADS1 (Kruskal-Wallis p=0.017). In WM, four elongases (ELOVL1 (Kruskal-Wallis p=0.0008, post-hoc Dunn’s test ADIII-IV vs MA p=0.001, ADV-VI vs MA p=0.0028), ELOVL4 (Kruskal-Wallis p=0.001, post-hoc Dunn’s test ADV-VI vs MA p=0.0026), ELOVL6 (post-hoc Dunn’s test ADIII-IV vs MA p=0.027, ADV-VI vs MA p=0.018) and ELOVL7 (Kruskal-Wallis p=0.003, post-hoc Dunn’s test ADIII-IV vs MA p=0.02, ADV-VI vs MA p=0.0009)) decreased its expression in AD progression and whereas FADS1 increases (post-hoc Dunn’s test ADV-VI vs MA p=0.0067). It is important to note that the effect of AD progression in mRNA FADS1 expression is common in both matters. Multivariate and univariate statistics were performed to analyze the 2,048 features of the whole lipidome. Multivariate statistics did not show global differences in the lipidome profile of the GM and the WM with AD progression (Fig. 5A and 5B).

**Table 4:** Fatty acid composition, general indexes, and estimated enzyme activities in the white matter (WM) in middle-aged individuals without NFTs and SPs (A) and in cases at AD stages I-II/0-A (B), III-IV/0-B (C), and V-VI/B-C (D).

Fatty acid	A	B	C	D	Kruskal-Wallis p-value	Kruskal-Wallis FDR p-value	Spearman’s Rho	Correlation p-value	Correlation corrected p-value
	*N=6*	*N=7*	*N=6*	*N=6*					
14:0	2.26 [2.06;2.40]	2.16 [1.93;2.36]	1.89 [1.46;2.08]	2.21 [2.06;2.33]	0.175	0.425	-0.144	0.491	0.783
16:0	14.9 [14.8;15.3]	15.4 [15.2;15.6]	16.4 [16.2;16.5]	15.6 [15.0;16.3]	0.194	0.43	0.293	0.153	0.482
16:1n-7	2.88 [2.75;3.02]	3.04 [2.84;3.13]	3.02 [2.96;3.07]	3.16 [3.11;3.31]	0.159	0.425	0.405	0.044	0.331
18:0	21.6 [21.3;22.2]	21.3 [21.2;21.4]	21.8 [21.2;22.0]	21.5 [21.2;21.7]	0.594	0.779	-0.110	0.598	0.811
18:1n-9	32.6 [31.8;33.2]	32.8 [32.4;33.6]	32.9 [32.5;33.0]	32.2 [31.9;32.4]	0.253	0.496	-0.159	0.447	0.735
18:1n-7	5.31 [4.78;5.57]	5.36 [4.96;5.64]	5.50 [5.43;5.77]	5.86 [5.63;6.37]	0.163	0.425	0.430	0.031	0.321
18:2n-6	0.26 [0.25;0.33]	0.28 [0.24;0.42]	0.38 [0.35;0.43]	0.25 [0.24;0.30]	0.236	0.481	0.048	0.819	0.889
18:3n-3	0.10 [0.08;0.11]	0.12 [0.11;0.14]	0.13 [0.11;0.14]	0.11 [0.11;0.12]	0.076	0.425	0.283	0.168	0.482
18:4n-3	0.43 [0.37;0.50]	0.44 [0.43;0.49]	0.47 [0.41;0.59]	0.51 [0.49;0.54]	0.328	0.532	0.366	0.071	0.331
20:0	0.28 [0.27;0.29]	0.28 [0.26;0.29]	0.26 [0.24;0.27]	0.29 [0.28;0.31]	0.173	0.425	0.087	0.677	0.825
20:1n-9	3.76 [3.28;3.83]	3.79 [2.83;4.15]	3.46 [3.15;3.50]	3.36 [2.94;3.61]	0.644	0.782	-0.262	0.205	0.524
20:2n-6	1.03 [0.86;1.11]	0.92 [0.88;0.94]	0.89 [0.89;0.96]	1.07 [0.97;1.21]	0.129	0.425	0.188	0.367	0.646
20:3n-3	0.68 [0.62;0.80]	0.63 [0.61;0.68]	0.55 [0.54;0.68]	0.61 [0.55;0.70]	0.473	0.67	-0.250	0.226	0.541
20:4n-6	2.90 [2.81;2.95]	2.76 [2.65;2.90]	2.95 [2.87;3.05]	2.81 [2.51;2.89]	0.334	0.532	-0.050	0.809	0.889
20:3n-6	0.25 [0.21;0.28]	0.20 [0.20;0.34]	0.22 [0.19;0.26]	0.21 [0.19;0.22]	0.602	0.779	-0.277	0.179	0.482
22:0	0.07 [0.07;0.08]	0.08 [0.07;0.09]	0.11 [0.08;0.11]	0.11 [0.10;0.11]	0.037	0.349	0.591	0.001	0.036
20:5n-3	0.48 [0.46;0.52]	0.41 [0.40;0.44]	0.45 [0.42;0.49]	0.51 [0.45;0.58]	0.367	0.567	0.060	0.774	0.877
22:1n-9	0.19 [0.18;0.22]	0.18 [0.17;0.19]	0.17 [0.16;0.18]	0.21 [0.18;0.23]	0.113	0.425	0.040	0.846	0.898
22:4n-6	3.29 [3.02;3.53]	2.97 [2.64;3.02]	2.88 [2.72;3.00]	2.76 [2.59;3.04]	0.108	0.425	-0.431	0.031	0.321
22:5n-6	0.28 [0.26;0.33]	0.19 [0.17;0.24]	0.23 [0.17;0.25]	0.22 [0.21;0.24]	0.089	0.425	-0.308	0.133	0.455
22:5n-3	0.09 [0.07;0.09]	0.10 [0.07;0.11]	0.11 [0.09;0.12]	0.09 [0.08;0.11]	0.434	0.651	0.236	0.254	0.541
24:0	1.01 [0.95;1.04]	0.85 [0.81;0.97]	0.76 [0.72;0.80]	0.90 [0.85;0.96]	0.041	0.349	-0.217	0.296	0.584
22:6n-3	0.71 [0.69;0.73]	0.77 [0.66;0.79]	0.89 [0.86;0.95]	0.77 [0.65;0.82]	0.015	0.293	0.192	0.357	0.646
24:1n-9	3.08 [2.95;3.26]	2.76 [2.57;3.01]	2.75 [2.46;3.09]	2.88 [2.75;3.73]	0.503	0.693	-0.035	0.865	0.900
24:5n-3	0.68 [0.47;0.87]	0.56 [0.52;1.32]	0.64 [0.51;1.01]	0.54 [0.51;0.58]	0.819	0.873	-0.159	0.447	0.735
24:6n-3	0.50 [0.43;0.54]	0.51 [0.39;0.54]	0.46 [0.40;0.54]	0.45 [0.44;0.57]	0.951	0.951	0.073	0.725	0.841
SFA	40.4 [40.0;41.5]	40.0 [39.9;40.8]	40.8 [40.5;41.0]	40.3 [39.8;41.6]	0.921	0.939	0.082	0.696	0.825
UFA	59.6 [58.5;60.0]	60.0 [59.2;60.1]	59.2 [59.0;59.5]	59.7 [58.4;60.2]	0.921	0.939	-0.082	0.696	0.825
PUFA	11.6 [11.5;12.1]	11.2 [11.0;12.2]	11.7 [11.4;11.9]	11.0 [10.6;11.2]	0.138	0.425	-0.333	0.103	0.400
MUFA	47.8 [46.8;48.6]	47.2 [46.8;48.9]	47.6 [47.3;48.1]	48.7 [47.3;49.5]	0.724	0.819	0.138	0.510	0.788
PUFAn-3	3.77 [3.66;4.21]	3.77 [3.38;4.34]	3.99 [3.76;4.18]	3.60 [3.58;4.02]	0.626	0.779	-0.106	0.611	0.811
PUFAn-6	7.85 [7.76;8.23]	7.29 [7.02;7.86]	7.55 [7.38;7.73]	7.37 [7.00;7.84]	0.457	0.666	-0.216	0.297	0.584
ACL	18.1 [18.1;18.1]	18.1 [18.0;18.2]	18.1 [18.0;18.2]	18.1 [18.0;18.1]	0.822	0.873	-0.104	0.620	0.811
DBI	95.1 [94.7;95.7]	93.5 [92.0;96.5]	94.7 [93.9;95.8]	92.3 [91.3;93.8]	0.102	0.425	-0.369	0.068	0.331
PI(a)	50.5 [48.6;52.6]	48.5 [46.8;52.4]	51.2 [49.4;51.6]	47.1 [44.7;47.9]	0.131	0.425	-0.327	0.109	0.400
PI(b)	36.5 [35.3;37.7]	34.9 [33.9;37.8]	36.8 [35.5;37.2]	34.0 [32.4;34.5]	0.135	0.425	-0.358	0.078	0.331
Δ9(n-7)	0.19 [0.18;0.20]	0.20 [0.18;0.20]	0.18 [0.18;0.19]	0.21 [0.19;0.21]	0.312	0.53	0.104	0.620	0.811
Δ9(n-9)	1.49 [1.43;1.56]	1.54 [1.49;1.59]	1.50 [1.48;1.55]	1.51 [1.50;1.52]	0.739	0.819	-0.022	0.915	0.934
Δ5(n-6)	10.3 [10.2;13.1]	13.2 [8.39;14.4]	13.5 [11.8;15.8]	13.9 [12.6;14.4]	0.614	0.779	0.239	0.248	0.541
Δ6(n-3)	5.13 [3.86;5.31]	3.61 [3.39;4.12]	3.65 [3.55;4.20]	4.49 [4.32;4.67]	0.186	0.43	0.002	0.989	0.989
Δ6(n-3)	0.69 [0.62;1.03]	0.65 [0.44;1.11]	0.68 [0.43;1.13]	0.94 [0.83;1.10]	0.714	0.819	0.189	0.365	0.646
Elovl3(n-9)	0.12 [0.10;0.12]	0.12 [0.09;0.12]	0.11 [0.10;0.11]	0.10 [0.09;0.11]	0.704	0.819	-0.237	0.252	0.541
Elovl6	1.45 [1.43;1.48]	1.39 [1.35;1.42]	1.32 [1.30;1.36]	1.38 [1.33;1.41]	0.146	0.425	-0.363	0.073	0.331
Elovl1-3-7a	0.01 [0.01;0.01]	0.01 [0.01;0.01]	0.01 [0.01;0.01]	0.01 [0.01;0.01]	0.097	0.425	0.132	0.528	0.792
Elovl1-3-7b	0.27 [0.26;0.27]	0.29 [0.28;0.31]	0.39 [0.35;0.43]	0.34 [0.31;0.38]	0.019	0.293	0.590	0.001	0.036
Elovl1-3-7c	14.2 [11.8;16.1]	11.4 [10.2;11.7]	8.07 [6.10;9.05]	8.77 [8.20;9.78]	0.022	0.293	-0.584	0.002	0.036
Elovl5(n-6)	3.72 [2.65;4.35]	3.20 [2.07;3.95]	2.48 [2.14;2.54]	4.51 [3.26;4.95]	0.206	0.438	0.082	0.694	0.825
Elovl2-5 (n-6)	1.12 [1.01;1.25]	1.08 [0.92;1.08]	1.00 [0.92;1.02]	1.00 [0.91;1.10]	0.279	0.508	-0.282	0.170	0.482
Elovl 2-5(n-3)	0.17 [0.12;0.20]	0.19 [0.18;0.22]	0.24 [0.21;0.27]	0.17 [0.14;0.23]	0.305	0.53	0.123	0.557	0.811
Elovl 2(n-3)	8.95 [8.34;9.39]	5.97 [5.33;20.0]	7.06 [6.16;8.30]	5.98 [5.06;6.91]	0.276	0.508	-0.387	0.055	0.331
Perox β-Ox	0.22 [0.20;0.23]	0.27 [0.23;0.28]	0.33 [0.29;0.35]	0.25 [0.23;0.28]	0.023	0.293	0.402	0.045	0.331

n: number of cases. Values are median [Q1;Q3] from 4-6 different individuals and are expressed as mole percent; p-values <0.05 are highlighted in bold. ACL: average chain length; SFA: saturated fatty acids; UFA: unsaturated fatty acids; MUFA: monounsaturated fatty acids; PUFA: polyunsaturated fatty acids; PUFAn-6: PUFA n-6 series; PUFAn-3: PUFA n-3 series; DBI: double-bond index; PI: peroxidizability index; NFTs: neurofibrillary tangle; SPs: senile plaques; FDR: False Discovery Rate. FDR was corrected for 51 tests.

For univariate statistics, we selected lipid species with a Kruskal-Wallis test p-value < 0.05; Spearman correlations were applied to identify molecules associated with disease progression (Tables 5 and 6, and Figures 5C and 5D). Results showed 50 statistically different (Kruskal-Wallis test p-value<0.05) lipid species in GM; 25 of them were identified based on exact mass, retention time, and/or MSMS spectrum (Table 5). Unknown lipid species are shown in Supplementary Table 5. Thus, in GM AD related lipid species are grouped into four major categories: i) Fatty acyls, 2 esters of fatty acids, FAHFA, and 1 AcylCoA, Retinoyl-CoA; ii) GLs, represented by 3 DG, 2 monoacylglycerols (MG), and 5 TG; iii) GP, including 3 PC, 2 PE, 1 PG, 1 PI, and 3 PS; and iv) Sphingolipids, represented by 1 sulfatide and 1 Cer. Only 6 specific lipid species correlated with AD progression: FAHFA(26:5) (Spearman’s Rho = -0.480, correlation p-value=0.023), TG(48:0) (Spearman’s Rho= -0.429, correlation p-value=0.045), TG(52:1) (Spearman’s Rho= -0.460, correlation p-value=0.031), PC(P-41:4) (Spearman’s Rho= -0.619, correlation p-value=0.002), PE(34:1) (Spearman’s Rho=0.516, correlation p-value=0.013) and PS(40:4) (Spearman’s Rho= -0.658, correlation p-value=0.0008) (Table 5).

**Table 5:** Significant distinctive lipidomic features in the frontal cortex (grey matter: GM) in middle-aged individuals without NFTs and SPs in any brain region (A), and in cases at Braak stages I-II/0-A (B), III-IV/0-B (C), and V-VI/B-C (D).

Class	Compound	Krukal-Wallis p-value	Krukal-Wallis FDR p-value	Post-hoc	Spearman’s Rho	Correlation p-value	Correlation FDR p-value	m/z value	RT
Fatty Acyls									
Fatty esters	FAHFA (24:3) a	0.042	0.845	A-C A-D	0.364	0.095	0.234	391.2854	3.66
	FAHFA (26:5) a	0.036	0.845	A-C B-C C-D	-0.480	0.023	0.134	415.3025	3.75
	Retinoyl CoA c	0.017	0.845	A-C B-C C-D	-0.185	0.408	0.505	1072.3095	7.62
Glycerolipids									
Diradylglycerols	DG(34:3) c	0.015	0.845	A-B A-D B-C C-D	-0.237	0.288	0.394	591.4963	8.1
	DG (38:5) c	0.029	0.845	A-D B-D C-D	-0.362	0.097	0.234	625.5211	6.85
	DG (44:4)) a	0.034	0.845	A-D B-D C-D	0.106	0.637	0.729	746.7144	7.16
Monoradylglycerols	MG (16:1) a	0.028	0.845	A-B B-D	0.267	0.228	0.376	346.332	2.6
	MG (18:1) a	0.045	0.845	A-B	0.271	0.221	0.376	374.3633	3.55
Triradylglycerols	TG (44:3) c	0.045	0.845	A-B A-C A-D	0.086	0.701	0.729	743.5977	7.92
	TG (48:0) a	0.035	0.845	A-C B-C C-D	-0.429	0.045	0.170	824.7713	10.14
	TG (50:0) a	0.014	0.845	A-C B-C C-D	-0.324	0.141	0.305	852.8003	10.31
	TG (52:1) a	0.025	0.845	A-C B-C C-D	-0.460	0.031	0.134	883.7737	10.33
	TG (54:4) a	0.035	0.845	B-D C-D	0.240	0.280	0.394	900.8038	10.03
Glycerophospholipids								
Glycerophosphocholines	PC(P-41:4) a	0.024	0.845	A-C A-D B-C B-D	-0.619	0.002	0.027	880.6352	7.91
	PC (O-42:2)/ PC (P-42:1) a	0.035	0.845	A-C A-D	-0.360	0.099	0.234	820.7033	7.3
	PC (40:5) a	0.032	0.845	A-D B-D C-D	0.057	0.800	0.800	836.6162	7.5
Glycerophosphoethanolamines	PE (34:1) a	0.032	0.845	A-B B-C B-D	0.516	0.013	0.120	718.572	7.32
	PE-Nme (36:2) a	0.032	0.845	A-D B-D C-D	-0.258	0.245	0.376	756.5448	7.51
Glycerophosphoglycerols	PG (37:0) c	0.037	0.845	A-B A-D C-D	0.090	0.689	0.729	827.5506	7.31
Glycerophosphoinositols	PI (34:1) a	0.032	0.845	A-D B-D C-D	-0.258	0.245	0.376	835.5253	6.11
Glycerophosphoserines	PS (36:0) a	0.011	0.845	A-D B-D C-D	0.088	0.695	0.729	792.5941	7.77
	PS (40:3) a	0.047	0.845	A-D B-D C-D	0.190	0.396	0.505	864.5726	7.42
	PS (40:4) a	0.017	0.845	A-C A-D B-C B-D	-0.658	0.0008	0.022	840.5738	6.79
Sphingolipids									
Acidic glycosphingolipids	Sulfatide (43:0) a	0.032	0.845	A-D B-D C-D	-0.258	0.245	0.376	906.6894	8.6
Ceramides	Cer(d48:2) a	0.046	0.845	A-D B-D	0.411	0.057	0.186	746.7042	7.18

Lipidomic features with p-value <0.05 after a Kruskal-Wallis test identified by exact mass, retention time, isotopic distribution, and MS/MS spectrum; Dunn test was used for Post-hoc analysis. Spearman’s correlation p-values <0.05 and Spearman’s correlation FDR p-values <0.05 are highlighted in bold; NFTs: neurofibrillary tangle; SPs: senile plaquesRT: retention time; FDR: False Discovery Rate. Kruskal-Wallis FDR p-values were corrected for 2,048 tests, and Spearman’s correlation FDR p-values were corrected for 25 tests.

However, only PC(P-41:4) (correlation FDR p-value=0.027) and PS (40:4) (correlation FDR p-value=0.022) correlations presented a corrected p-value < 0.05. Regarding the WM, 90 lipid species were statistically different (p<0.05), and 57 of them were identified based on the criteria defined above (Table 6 and Supplementary Table 6 for details on unknown lipid species). Different lipid species were grouped into five major categories: i) Fatty acyls: 4 esters of fatty acids as FAHFAs, and 1 Acyl carnitine, the clupanodonyl carnitine; ii) GLs: 4 DG, 1 MG, and 2 TG; iii) GP: 8 PC, 10 PE, 1 CL, 1 PI, 2 PS, and the phosphorylcholine, iv) Sphingolipids, was comprised of 8 Cer, 10 HexCer, 1 sulfatide, and 2 SM; and v) Sterol lipids: 1 CE (Table 6). Changes of 38 molecules in the WM correlated with AD’s progression, 36 with a corrected p-value<0.05. Among them, we found 1 FAHFA with a positive correlation with disease progression (FAHFA (23:0), Spearman’s Rho=0.501, correlation p-value=0.01) and another FAHFA (FAHFA (38:6), Spearman’s Rho=-0.513, correlation p-value=0.008) and the clupanodonyl carnitine (Spearman’s Rho=-0.492, correlation p-value=0.012) with a negative correlation. Three of four DG (DG(34:1), Spearman’s Rho=-0.485, correlation p-value=0.013; DG(38:1), Spearman’s Rho=-0.613, correlation p-value=0.001; DG(40:4), Spearman’s Rho=-0.534, correlation p-value=0.005) and 2 TG (TG(40:1), Spearman’s Rho=-0.485, correlation p-value=0.013; TG(46:1), Spearman’s Rho=-0.556, correlation p-value=0.003) decreased, whereas the MG(13:0) (Spearman’s Rho=0.488, correlation p-value=0.013) increased with disease progression. GP identified 9 molecules negatively and 5 positively correlated with AD progression. Remarkably, 5 were ether lipids (PC(P-32:1), Spearman’s Rho=-0.526, correlation p-value=0.006; PC(P-41:3), Spearman’s Rho=-0.671, correlation p-value=0.0002; PE(P-38:3), Spearman’s Rho=0.52, correlation p-value=0.007; PE(P-38:4), Spearman’s Rho=´-0.6, correlation p-value=0.001; PE(P-40:4), Spearman’s Rho=-0.529, correlation p-value=0.006), and 1 was an oxidized form of PE (oxPE(16:2), Spearman’s Rho=0.513, correlation p-value=0.008). Finally, 15 of the 21 differentially expressed sphingolipids correlated with AD progression. Interestingly, 14 of these 15 (93.75%) and 12 of the 13 (92.3%) with a corrected p-value < 0.05 -including sulfatides, ceramides, and glycosphingolipids- correlated negatively with disease progression (Table 6). Only 2 differential lipid species were common between GM and WM, the DG (38:5) and the Cer (d48:2). These results indicate that sAD affects differentially both brain matters and that the changes are more marked in WM.

Since a relevant number of lipid species correlating with AD progression were ether lipids synthesized in peroxisomes, we also analyzed the mRNA expression levels of selected genes linked to peroxisomal import of fatty acids, peroxisomal β-oxidation and peroxisomal biogenesis (Fig. 4). The analyses of peroxisomal-related genes showed that acids ATP-binding cassette transporters of subfamily D (ABCD) revealed that the expression of genes affected is down-regulated in AD. Specifically, in GM we found decreased levels of ABCD2 (Kruskal-Wallis p=0.01, post-hoc Dunn’s test ADIII-IV vs MA p=0.005, ADV-VI vs MA p=0.02) and ABCD3 (post-hoc Dunn’s test ADIII-IV vs MA p=0.02) whereas in WM the genes affected were ABCD1 (post-hoc Dunn’s test ADI-II vs MA p=0.03) and, as it happened in GM, the ABCD3 (Kruskal-Wallis p=0.0097, post-hoc Dunn’s test ADIII-IV vs MA p=0.009). Additionally, mRNA expression of peroxisomal β-oxidation related genes were evaluated. In GM, although no global changes were observed, the expression levels of ACOX1 and ACOX3 decreased in stages V-VI (post-hoc Dunn’s test p=0.01) and III-IV (post-hoc Dunn’s test p=0.04), respectively, when compared with MA individuals. In WM we observed more global changes including decreased levels of ACAA1 (Kruskal-Wallis p=0.0001, post-hoc Dunn’s test ADI-II vs MA p=0.0009, ADIII-IV vs MA p=0.00001, and ADV-VI vs MA p=0.002) and EHHDAH (post-hoc Dunn’s test ADIII-IV vs MA p=0.03, and ADV-VI vs MA p=0.04), and increased levels of ACOX3 (Kruskal-Wallis p=0.03, post-hoc Dunn’s test ADI-II vs MA p=0.003, and ADV-VI vs MA p=0.01), reinforcing the idea that AD affects differently both brain matters.

Peroxisomal biogenesis genes expression decreases in GM and increases in WM, reinforcing the different affectation of AD in crucial mechanisms and pathways depending on the brain location. Specifically, in GM the PPARG (post-hoc Dunn’s test ADIII-IV vs MA p=0.004), PPARGC1 (Kruskal-Wallis p=0.01, post-hoc Dunn’s test ADIII-IV vs MA p=0.0006, and ADV-VI vs MA p=0.04) and DNML1 (Kruskal-Wallis p=0.02, post-hoc Dunn’s test ADIII-IV vs MA p=0.02, and ADV-VI vs MA p=0.02) were affected whereas the PPARD (Kruskal-Wallis p=0.005, post-hoc Dunn’s test ADIII-IV vs MA p=0.002, and ADV-VI vs MA p=0.002), PPARG (Kruskal-Wallis p=0.01, post-hoc Dunn’s test ADV-VI vs MA p=0.049) and PPARGC1(post-hoc Dunn’s test ADIII-IV vs MA p=0.008) were affected in WM. Finally, the importance of FAHFA species in our study led us to evaluate the mRNA expression of the FAHFA biosynthesis related gene bile acid CoA: amino acid N-acyltransferase (BAAT). The results showed that BAAT increased in middle-latter stages of AD only in WM (Kruskal-Wallis p=0.01, post-hoc Dunn’s test ADIII-IV vs MA p=0.001, and ADV-VI vs MA p=0.01).

**Table 6:** Significant distinctive lipidomic features in the white matter (WM)in middle-aged individuals without NFTs and SPs in any brain region (A), and cases at Braak stages I-II/0-A (B), III-IV/0-B (C), and V-VI/B-C (D).

Class	Compound	Krukal-Wallis p-value	Krukal-Wallis FDR p-value	Post-hoc	Spearman’s Rho	Correlation p-value	Correlation FDR p-value	m/z value	RT
Fatty Acyls									
Fatty esters	FAHFA (20:0) a	0.027	0.801	A-B A-C	0.312	0.128	0.161	341.2656	0.93
	FAHFA (23:0) a	0.036	0.801	A-C B-C C-D	0.501	0.010	0.025	383.2761	0.93
	FAHFA (23:3) a	0.032	0.801	A-B A-C C-D	-0.212	0.308	0.337	377.2691	3.68
	FAHFA (38:6) a	0.004	0.801	A-C B-C C-D	-0.513	0.008	0.022	581.4277	6.74
	Clupanodonyl carnitine b	0.010	0.801	A-C B-C C-D	-0.492	0.012	0.028	512.3154	7.46
Glycerolipids									
Diradylglycerols	DG (34:1) c	0.033	0.801	A-B B-C C-D	-0.485	0.013	0.028	577.5203	7.22
	DG (38:1) a	0.013	0.801	A-B B-C C-D	-0.613	0.001	0.013	668.6557	8.44
	DG (38:5) c	0.020	0.801	A-C B-C C-D	-0.285	0.167	0.189	625.5211	6.85
	DG (40:4) c	0.033	0.801	B-C C-D	-0.534	0.005	0.022	655.5657	6.77
Monoradylglycerols	MG (13:0) c	0.044	0.801	A-C A-D B-C	0.488	0.013	0.028	269.2096	0.92
Triradylglycerols	TG (40:1) a	0.027	0.801	A-C A-D B-C	-0.485	0.013	0.028	710.5937	9.24
	TG(46:1) a	0.032	0.801	A-C B-C	-0.556	0.003	0.022	794.6884	8.42
Glycerophospholipids									
Glycerophosphocholines	PC (P-32:1) a	0.049	0.801	A-C B-C C-D	-0.526	0.006	0.022	760.5204	7.25
	PC (O-44:6) a	0.040	0.801	A-B A-C	0.032	0.878	0.893	858.6796	8.13
	PC(O-41:4)/PC(P-41:3) a	0.004	0.801	A-C A-D B-C	-0.671	0.0002	0.009	882.6903	8.38
	PC(P-42:1)/PC(O-42:2) a	0.035	0.801	A-C B-D C-D	-0.100	0.634	0.656	856.6781	8.88
	PC(34:3) a	0.011	0.801	A-C C-D	-0.305	0.137	0.166	756.5556	6.69
	PC(42:8) a	0.046	0.801	A-C B-C C-D	-0.505	0.010	0.024	858.6028	6.85
	PC(44:4) a	0.008	0.801	A-C A-D B-C B-D	-0.659	0.0003	0.009	894.6818	8.66
	LPC(18:0) a	0.047	0.801	A-C C-D	-0.359	0.077	0.106	546.4002	0.92
Glycerophosphoethanolamines	PE(P-38:3) a	0.030	0.801	A-C B-C	0.520	0.007	0.022	752.543	6.26
	PE(P-38:4) a	0.010	0.801	A-C B-C C-D	-0.600	0.001	0.013	752.5611	7.49
	PE(P-40:4) a	0.024	0.801	A-C B-C	-0.529	0.006	0.022	780.5926	7.87
	PE(P-42:0) c	0.047	0.801	A-C C-D	-0.308	0.133	0.163	796.6574	8.07
	PE(P-38:2)/PE(O-38:3) a	0.011	0.801	A-B A-C A-D	-0.133	0.523	0.551	754.6103	7.65
	oxPE(16:2) b	0.020	0.801	A-C B-C C-D	0.513	0.008	0.022	506.224	2.68
	PE(29:0) a	0.047	0.801	A-C C-D	-0.290	0.158	0.186	650.4968	4.41
	PE(44:0) c	0.045	0.801	A-B B-C B-D	0.375	0.064	0.090	894.6527	8.13
	PE-Nme2(44:8) c	0.018	0.801	A-D B-D C-D	-0.164	0.430	0.462	872.6015	8.04
	LPE(20:1) a	0.029	0.801	A-C B-C C-D	-0.536	0.005	0.022	508.341	0.92
Glycerophosphoglycero-phosphoglycerols	CL(72:5) b	0.038	0.801	A-C B-C	0.453	0.022	0.040	726.5268	6.18
Glycerophosphoinositols	PI(40:6) c	0.029	0.801	A-C A-D B-C	0.519	0.007	0.022	909.5395	6.16
Glycerophosphoserines	PS(40:5) a	0.032	0.801	A-C B-C C-D	-0.519	0.007	0.022	838.5583	6.69
	PS(42:4) c	0.028	0.801	A-D B-D	0.443	0.026	0.042	902.5922	6.51
Other	Phosphorylcholine b	0.045	0.801	B-C	-0.552	0.004	0.022	184.0729	8.35
Sphingolipids									
Acidic glycosphingolipids	Sulfatide(39:4) a	0.009	0.801	A-C A-D B-D	-0.480	0.014	0.029	842.5798	7.18
Ceramides	Cer(d41:2) a	0.014	0.801	A-C B-C C-D	-0.409	0.042	0.062	634.615	8.13
	Cer(d48:2) a	0.037	0.801	A-B B-C C-D	0.517	0.008	0.022	746.7042	7.18
	Cer(d17:2) c	0.033	0.801	A-C B-C	-0.443	0.026	0.042	438.4316	6.64
	Cer(d28:2) c	0.044	0.801	A-C B-C C-D	-0.473	0.016	0.030	496.4319	6.63
	Cer(d40:0) a	0.048	0.801	A-C A-D	-0.375	0.064	0.090	624.6296	8.19
	Cer(d42:1) a	0.007	0.801	A-C A-D B-C B-D	-0.593	0.001	0.013	632.6325	8.44
	OxCer(40:1) a	0.033	0.801	A-B A-D B-C C-D	-0.324	0.113	0.147	644.5988	7.61
	Cer(d44:1) a	0.008	0.801	A-B A-C C-D	-0.415	0.039	0.059	660.6653	9.18
Neutral glycosphingolipids	HexCer(d36:1) c	0.020	0.801	A-C B-C C-D	-0.520	0.007	0.022	872.6422	6.92
	HexCer(d40:1) a	0.020	0.801	A-C B-C C-D	-0.447	0.025	0.042	766.6576	8.05
	HexCer(d40:2) a	0.022	0.801	A-C B-C C-D	-0.528	0.006	0.022	782.6517	7.92
	HexCer(d40:3) a	0.033	0.801	A-C B-C B-D	-0.598	0.001	0.013	780.6729	8.24
	HexCer(d41:2) a	0.047	0.801	A-C B-C C-D	-0.478	0.015	0.029	796.6687	8.13
	HexCer(d42:3) a	0.005	0.801	A-C A-D B-C B-D	-0.636	0.0006	0.012	808.7043	8.58
	HexCer(d43:1) c	0.029	0.801	A-B A-C A-D	-0.340	0.095	0.128	824.6892	8.6
	HexCer(d43:2) a	0.013	0.801	A-C B-C C-D	0.009	0.963	0.963	824.7002	8.49
	HexCer(d43:2) a	0.038	0.801	A-D B-D C-D	-0.605	0.001	0.013	824.6959	7.11
	HexCer (t43:1) a	0.016	0.801	A-C B-C C-D	-0.559	0.003	0.022	856.6795	8.43
Phosphosphingolipids	SM (d28:5) c	0.015	0.801	A-B B-D	0.323	0.114	0.147	655.4136	6.22
	SM (d35:1) c	0.041	0.801	A-B A-C	-0.281	0.173	0.193	715.5661	7.32
Sterol Lipids									
Sterols	CE (18:1) c	0.019	0.801	A-C C-D	-0.289	0.161	0.186	633.5821	8.83

Lipidomic features with p-value <0.05 after a Kruskal-Wallis test identified by exact mass, retention time, isotopic distribution, and MS/MS spectrum; Dunn test was used for Post-hoc analysis. Spearman’s correlation p-values <0.05 and Spearman’s correlation FDR p-values <0.05 are highlighted in bold; NFTs: neurofibrillary tangle; SPs: senile plaquesRT: retention time; FDR: False Discovery Rate. Kruskal-Wallis FDR p-values were corrected for 2,048 tests, and Spearman’s correlation FDR p-values were corrected for 57 tests.

### WM and GM lipidome changes linked to AD progression

Among the 25 identified lipids associated with AD progression in GM, 9 (36%) coincided with differential lipids between GM and WM. Seven of them (78%) were up-regulated in GM. Among the 57 identified lipids associated with AD progression in WM, 25 (42%) coincided with differential lipids between WM and GM. Twenty-one (83%) were up-regulated in the WM compared with GM.

## DISCUSSION

This study was designed to learn about WM and GM lipidomic modifications in MA individuals without NFT and SP as primary markers of AD-related pathology and cases at progressive stages of sAD. Special care was taken to exclude cases with co-morbidities and associated pathologies.

In individuals with no lesions, the WM is characterized by enrichment in MUFA, particularly oleic acid, 18:1n-9, and decreased content of SFA, PUFA, PUFAn-3, and PUFAn-6 resulting in a lower DBI and PI when compared with the GM. This lipid configuration is accompanied by higher delta 9-desaturase and elongase activities and decreased activity of delta-5 and delta-6 desaturases in the WM. Interestingly, both regions maintain the average chain length of 18 carbon atoms. Previous studies -covering a limited range of fatty acids- also showed similar differences in fatty acid profiles between WM and GM [35, 70, 90, 91]. In addition, the analysis of the whole lipidome in the present study demonstrates a lower concentration of DGs, PAs, and CEs, and significant enrichment in TG, PC, PE, sulfatides, ceramides, glycosphingolipids, and sphingomyelins in the WM compared with GM. Our results are in line and go further from earlier studies showing differences in lipid composition between GM and WM [70]. Differences in the lipid composition between GM and WM have also been identified in the frontal lobe using MALDI-TOF mass spectrometry-imaging [92] and flow infusion analysis coupled to Orbitrap™ mass spectrometry [93] in other brain regions as the temporal lobe [94] and the caudate nucleus [95]. Among the differential lipids in WM, we show the enrichment in lipid species belonging to the ether lipid class (alkyl and alkenyl ethers) mostly presented as TG, PC, and PE species. Considering this scenario, we demonstrate that the WM, with a higher content of UFA but with a lower degree of unsaturation, maintains the geometry and the physicochemical properties of cell membranes determining lower susceptibility to oxidative damage (lower PI), along with a higher antioxidant property linked to the high content of ether lipids. These properties constitute a more resistant condition to lipid peroxidation and, consequently, a protective environment for axonal projections.

Seminal studies reported reduced galactosylceramide (GalCer) and sulfatide, and increased cholesterol and fatty acid contents in both GM and WM in AD [96-100]. Levels of GalCer and sulfatide - synthesized by oligodendrocytes and major myelin components - slightly decrease in the frontal and temporal cortex and WM at stages III-IV and more markedly at stages V-VI in AD [101]. Interestingly, the activity of ceramide synthase 2, the enzyme that catalyzes the synthesis of very-long-chain ceramides, decreases in the temporal cortex at early stages and in the frontal cortex at middle-advanced stages, thus showing that alterations of ceramide synthesis occur in the early stages of AD [101]. We also observed decreased levels of specific phospholipid components of myelin in AD and reduced expression of myelin-associated proteins at advanced stages of sAD [31].

Fatty acids are inherent components of GLs, GPs, and sphingolipids. The number of carbon atoms and double bonds determines the geometric traits of lipids influencing membrane organization and function [102]. Besides, fatty acids are substrates for the generation of lipid signaling mediators, particularly relevant for PUFAn-6 and PUFAn-3 [103]. An additional trait assigned to fatty acids is their chemical reactivity in oxidative conditions determining the susceptibility to oxidative damage for a given membrane [104]. Oxidant agents attack PUFA side chains much more easily than SFA and MUFA side chains (this fact is expressed by DBI and PI parameters). Our results show that sAD progression in the GM is associated with an enrichment in PUFA, particularly 20:4n-6 and 22:6n-3, leading to a fatty acid profile more prone to lipid peroxidation, consistent with previous data [61]. Regarding the WM, we observed increased levels of 16:1n-7, 18:1n-9, 22:0, and 22:4n-6 (with no changes in DBI and PI indexes). Furthermore, the changes in mRNA expression of desaturases and elongases in sAD is clearly different in both matters. The dissociation between genotype and lipotype suggests additional post-transcriptional changes and mechanisms (e.g., diacylation/reacylation and/or oxidative damage) in the determination of fatty acid profile.

All in all, these results indicate that minor but significant changes in the fatty acids profile mainly occur in the GM with AD progression, associated with significant vulnerability to oxidative stress conditions favored by the peroxidation-prone membrane lipid profile. By contrast, changes in fatty acid profile in the WM are associated with peroxidation-resistant membranes. Thus, we suggest that changes in GM have a clear deleterious effect, while in WM play a protective role likely as adaptative respond induced by AD pathology.

The global lipidome analyses show that the number of lipid species linked with AD progression is higher in WM than in GM. Thus, levels of 90 lipid species are statistically different comparing MA individuals and AD stages. Among 57 differential molecules with a potential identity, 38 correlated with AD progression (36 with a correlation FDR p-value < 0.05). Specifically, decrease of ceramides and hexosylceramides with AD progression were observed and two FAHFA species showed association with AD progression. In contrast, in the GM, 51 lipid species showed different levels in MA and AD stages, 25 of them with a potential identity; levels of 6 lipids correlate with AD progression. Specifically, in GM the changes include a decrease in the concentration of FAHFA, TG, PC plasmalogens, and PS, together with enrichment in PE and ceramides with AD progression.

FAHFA, derived from the activity of patatin-like phospholipase domain containing 2 (PNPLA2, also known ATGL) [105], are involved in glucose homeostasis, insulin resistance and anti-inflammatory functions. FAHFA are also linked to the nuclear factor erythroid 2-related factor 2 (Nrf2), which participates in antioxidant cell defenses [106, 107]. Of note, no previous data were available on the implication of these mediators in AD, but due to the implication of insulin resistance as a risk factor for AD [108] it might be hypothesized that local FAHFA metabolism could play a patho-physiological role in AD. Specifically, our work demonstrated the implication of FAHFA species in AD progression in both GM and WM. In order to further elucidate the implication of FAHFA metabolism in AD progression, we also analyzed the mRNA expression of BAAT gene, previously related with FAHFA biosynthesis [107] that showed a significant increase with AD progression in WM. All in all, the results suggest that FAHFA metabolism could be important in AD physiopathology.

DG and TG belong to the GL category. DG are cell membrane components and intermediates of lipid metabolism and act as second messengers modulating transduction proteins such as protein kinases [109-111]. Previous studies have shown altered DG levels [112, 113] and deregulated protein phosphorylation [114] in AD. A global decrease of DG levels more marked in WM may contribute to altered membrane signaling and altered protein phosphorylation in sAD [114]. Moreover, TG are bioenergetic compounds that, along with cholesteryl esters, are components of lipid droplets in neural cells [115]. The decrease of TG in GM and WM suggests an adaptive response to higher neuronal bioenergetic demands with AD progression [116].

GP are integral components of cell membranes, substrates forming second messengers and lipid mediators, and targets and sources of oxidative stress. GP participates in a wide diversity of cell mechanisms involved in cell proliferation and differentiation, autophagy, and synthesis of other GP classes [36, 102, 117]. Reduced GP levels occur in aging and neurodegeneration [56]. Our study shows GP down-regulation, mainly affecting PC and PE levels, with AD progression, thus suggesting alterations in the architecture of neural cell membranes.

Ether lipids are a subclass of GP that show two chemical forms: alkyl ethers and alkenyl ethers or plasmalogens [118, 119]. Ether lipids are primarily present as PC and PE species but have also been described as TG [119]. Their biosynthesis begins in the peroxisome and is completed in the endoplasmic reticulum [119, 120]. The physiological role of ether lipids is essentially associated with their function as membrane components with antioxidant properties [121]. Lower ether lipid content is negatively associated with cancer, cardiovascular diseases, and AD [122, 123]. The present study reveals a down-regulation of ether lipids in GM and, more markedly, in WM with AD progression. Consistent with these observations, there is an increase in oxidized PE in WM with AD progression, in agreement with enhanced lipoxidation reactions in AD [61]. Based on these observations, the lipidomic results were orthogonally validated analyzing the mRNA expression levels of selected genes linked to peroxisomal import of fatty acids, peroxisomal β-oxidation and peroxisomal biogenesis.

Firstly, the mRNA expression of peroxisomal import of fatty acids related genes globally decreases in both matters, suggesting a general decrease of this mechanism in AD progression. Secondly, peroxisomal β-oxidation related genes revealed slightly changes in GM during AD progression whereas WM is more affected. Finally, peroxisomal biogenesis seems to be decreased in GM and increased in WM in AD progression, changes probably orchestrated by PPARs. The PPARs are nuclear receptors that function as ligand-activated transcription factors that regulate numerous biological processes like metabolism of glucose and lipids, inflammation, cellular differentiation, and proliferation [124]. Interestingly, PPARs have been previously related to neurodegenerative disorders [125] and Aβ production [126, 127]. Although the role of PPARs in the brain has been mainly related to lipid metabolism, these receptors are also implicated in neural cell differentiation and death, inflammation, and neurodegeneration [128]. Furthermore, PPARs can also exert protective activity against oxidative damage, inducing the expression of antioxidant enzymes [129]. The different up- or down-regulation of these nuclear receptors and its coactivator that we find in both matters could be mediating greater protection in the WM while the GM would be more exposed to neuronal damage. These results agree with previous data suggesting the implication of PPAR in AD pathogenesis [130]. The contradiction between mRNA expression and down-regulation of ether lipids may result from a peroxisomal adaptation to an increased consumption rate or damage of ether lipids due to AD-induced oxidative stress conditions. And indeed, mRNA expression levels do not necessarily parallel protein levels encoded by the corresponding genes.

Sphingolipids constitute a complex lipid group derived from N-acylsphingosine (ceramide), which is highly expressed in the human brain [35]. This chemical group includes a broad diversity of lipid species with structural and bioactive/messenger functions that play a vital role in the composition of lipid rafts [131, 132]. Sphingolipids regulate membrane physiology and cell biology (e.g., oxidative stress, apoptosis, and cell survival); they are involved in pathological conditions such as cardiovascular diseases and neurodegeneration [41, 64, 132-136]. Our lipidomic study shows limited sphingolipid alterations in GM but a higher content of sphingolipids in WM in AD. In WM, 15 lipid species correlate with AD progression. Only 1 lipid species (Cer(d48:2) positively correlates, while 14 negatively correlate with AD progression. These results agree with recently described alterations of sphingolipid metabolism in AD, related to amyloid precursor protein-induced changes in the mitochondria-endoplasmic reticulum communications [137].

Regarding other lipid species, sulfatides, ceramides, and glycosphingolipids are decreased with AD progression, thus suggesting a negative impact on lipid raft structure and function. Lipid rafts are membrane microdomains that facilitate intercellular interactions through membrane ion channels and various signaling receptors. Membrane proteins and components of the cytoskeleton anchor in and bind to lipid rafts and regulate receptor activation, signaling pathways, membrane protein trafficking, neurotransmission, cytoskeleton, and cellular polarity [138]. Our findings point to alterations in lipid rafts composition since two major components, sphingolipids and phospholipids are affected in AD. Present findings are in line with previous observations showing altered lipid composition of lipid rafts in the brain aging and AD [41, 64, 139, 140]. Moreover, experimental pieces of evidence point to the facilitation of β-amyloid production resulting from abnormalities in the lipid raft composition in sAD [141-143].

It is well described that the incidence of AD is significantly different between males and females [144] and that the lipidome of healthy and pathological individuals is differentially affected by gender [145], suggesting that the effect of AD on the lipidome could also be different in males than in females. Due to limitations in the sample size of the study, this approach could not be performed, and further studies should focus on this aspect.

In summary, the present study characterizes the lipidome in the GM of the frontal cortex and WM of the frontal lobe *centrum semi-ovale* in MA individuals and at progressive stages of AD. The results indicate that WM is characterized by a fatty acid profile resistant to lipid peroxidation and by an enrichment of various lipid classes, mainly ether lipids, compared to GM. Furthermore, AD progression implicates a modulation of the lipidomic profile, with an impact in the main lipid categories implicated in cellular membranes structure, bioenergetics, antioxidant protection and cell signaling. Importantly, this lipidomic modulation had a higher impact on WM. In particular, WM in AD is characterized by a progressive decline in the content of DG, TG, GP (especially ether lipids), and sphingolipids (especially sulfatides, ceramides, and glycosphingolipids), and with specific changes in the metabolism of FAHFA.

## Supplementary Materials

The Supplementary data can be found online at: www.aginganddisease.org/EN/10.14336/AD.2023.0217.



## Data Availability

The datasets generated and/or analyzed during the current study are available from the corresponding author upon reasonable request.
