# Supplementary material for: Lipidomic Alterations in the Cerebral Cortex and White Matter in Sporadic Alzheimer’s Disease
Source: Aging Dis. 2023 Oct 1;14(5):1887–916. doi: 10.14336/AD.2023.0217 (PMC10529741; doi:10.14336/AD.2023.0217)
Supplement: Supplementary file 1 [file AD-14-5-1887-s.pdf]

SUPPLEMENTARY DATA

Lipidomic Alterations in the Cerebral Cortex and White Matter in Sporadic Alzheimer's Disease

Elia Obis[#], Joaquim Sol[#], Pol Andres-Benito, Meritxell Martín-Gari, Natàlia Mota-Martorell, José Daniel Galo-Licona, Gerard Piñol-Ripoll, Manuel Portero-Otin, Isidro Ferrer, Mariona Jové[*], Reinald Pamplona[*]

© 2023. Obis E et al. Published online at http://www.aginganddisease.org/EN/10.14336/AD.2023.0217

SUPPLEMENTARY DATA

Supplementary Table 1. Class representative and extraction internal standards added to the samples in untargeted lipidomics analysis.

COMPOUND	SOURCE	IDENTIFIER
**1,3(d5)-dihexadecanoyl-glycerol**	Avanti Polar Lipids	110537
**1,3(d5)-dihexadecanoyl-2-octadecanoyl-glycerol**	Avanti Polar Lipids	110543
**1-hexadecanoyl(d31)-2-(9Z-octadecenoyl)-sn-glycero-3-phosphate**	Avanti Polar Lipids	110920
**1-hexadecanoyl(d31)-2-(9Z-octadecenoyl)-sn-glycero-3-phosphocholine**	Avanti Polar Lipids	110918
**1-hexadecanoyl(d31)-2-(9Z-octadecenoyl)-sn-glycero-3-phosphoethanolamine**	Avanti Polar Lipids	110921
**1-hexadecanoyl-2-(9Z-octadecenoyl)-sn-glycero-3-phospho-(1'-rac-glycerol-1',1',2',3',3'-d5)**	Avanti Polar Lipids	110899
**1-hexadecanoyl(d31)-2-(9Z-octadecenoyl)-sn-glycero-3-phospho-myo-inositol**	Avanti Polar Lipids	110923
**1-hexadecanoyl(d31)-2-(9Z-octadecenoyl)-sn-glycero-3-[phospho-L-serine]**	Avanti Polar Lipids	110922
**26:0-d4 Lyso PC**	Avanti Polar Lipids	860389
**18:1 Chol (D7) ester**	Avanti Polar Lipids	111015
**cholest-5-en-3ß-ol (d7)**	Avanti Polar Lipids	LM-4100
**D-erythro-sphingosine-d7**	Avanti Polar Lipids	860657
**D-erythro-sphingosine-d7-1-phosphate**	Avanti Polar Lipids	860659
**N-palmitoyl-d31-D-erythro-sphingosine**	Avanti Polar Lipids	868516
**N-palmitoyl-d31-D-erythro-sphingosylphosphorylcholine**	Avanti Polar Lipids	868584
**Octadecanoic acid-2,2-d2**	Sigma Aldrich	19905-58-9

Supplementary Table 2. Gene symbols and Taqman probes used for RT-qPCR.

Gene	Full name	Taqman probe
ABCD1	ATP Binding Cassette Subfamily D Member 1	Hs00163610_m1
ABCD2	ATP Binding Cassette Subfamily D Member 2	Hs00193054_m1
ABCD3	ATP Binding Cassette Subfamily D Member 3	Hs00161065_m1
ACAA1	Acetyl-CoA Acyltransferase 1	Hs01576070_m1
BAAT	Bile Acid-CoA:Amino Acid N-Acyltransferase	Hs00156051_m1
EHHADH	Enoyl-CoA Hydratase And 3-Hydroxyacyl CoA Dehydrogenase	Hs00157347_m1
GUS-β	β-glucuronidase	Hs00939627_m1
PPARA	Peroxisome Proliferator Activated Receptor Alpha	Hs00947539_m1
PPARD	Peroxisome Proliferator Activated Receptor Delta	Hs00606407_m1
PPARG	Peroxisome Proliferator Activated Receptor Gamma	Hs01115513_m1
PPARGC1A	PPARG Coactivator 1 Alpha	Hs00173304_m1
ELOVL1	Elongation Of Very Long Chain Fatty Acids Protein 1	Hs00249277_m1
ELOVL2	Elongation Of Very Long Chain Fatty Acids Protein 2	Hs00214936_m1
ELOVL3	Elongation Of Very Long Chain Fatty Acids Protein 3	Hs00537016_m1
ELOVL4	Elongation Of Very Long Chain Fatty Acids Protein 4	Hs00224122_m1

© 2023. Obis E et al. Published online at http://www.aginganddisease.org/EN/10.14336/AD.2023.0217

ELOVL5	Elongation Of Very Long Chain Fatty Acids Protein 5	Hs01094711_m1
ELOVL6	Elongation Of Very Long Chain Fatty Acids Protein 6	Hs00225412_m1
ELOVL7	Elongation Of Very Long Chain Fatty Acids Protein 7	Hs00405151_m1
ACOX1	Peroxisomal Acyl-Coenzyme A Oxidase 1	Hs01074241_m1
ACOX2	Peroxisomal Acyl-Coenzyme A Oxidase 2	Hs00185873_m1
ACOX3	Peroxisomal Acyl-Coenzyme A Oxidase 3	Hs01089970_m1
DNM1L	Dynamin 1 Like	Hs00247147_m1
FADS1	Fatty Acid Desaturase 1	Hs01096545_m1
FADS2	Fatty Acid Desaturase 2	Hs00927433_m1

Supplementary Table 3. Identified **s**ignificant distinctive lipidomic features for white and grey matter in brain tissue.

Class	Compound	Mann-Whitney p-value	Mann-Whitney FDR p-value	Regulation (WM vs GM)	m/z value	Retention time
**Fatty Acyls**						
**Fatty esters**	FAHFA(34:1;O) c	0.0095238	0.0667	down	556.5378	8.1
	FAHFA(19:0) a	0.0095238	0.0667	down	327.2299	2.7
	FAHFA(43:4) a	0.0095238	0.0667	down	655.5557	8.2
	FAHFA(47:5) a	0.0095238	0.0667	up	709.6028	8.5
	FAHFA(45:3) a	0.0095238	0.0667	up	685.584	8.5
	FAHFA(48:5) a	0.0095238	0.0667	up	723.6176	8.7
	Retinoyl CoA c	0.0095238	0.0667	down	1072.3095	7.6
**Glycerolipids**						
**Diradylglycerols**	DG(36:4) c	0.0057411	0.0667	down	627.5349	7.3
	DG(40:5) c	0.0057411	0.0667	down	653.5509	7.5
	DG(38:7) c	0.0057411	0.0667	down	635.5006	8.5
	DG(38:6) c	0.0089113	0.0667	down	623.5045	6.7
	DG(36:4) c	0.0089113	0.0667	down	599.5071	8.9
	DG(36:1) c	0.0095238	0.0667	up	605.5511	6.8
	DG(36:2) c	0.0095238	0.0667	up	603.5352	7.3
	DG(40:4) c	0.0095238	0.0667	down	655.5665	7.6
	DG(32:2) b	0.0095238	0.0667	down	596.5299	8.1
	DG(34:3) c	0.0095238	0.0667	down	591.4963	8.1
	DG(34:1) c	0.0095238	0.0667	up	577.5195	8.1
	DG(38:1) a	0.0095238	0.0667	up	668.6557	8.4
	DG(32:1) a	0.0095238	0.0667	down	584.5668	8.5
	DG(36:3) c	0.0095238	0.0667	down	619.5278	8.5
	DG(40:6) c	0.0095238	0.0667	up	686.5863	8.5
**Triradylglycerols**	TG(O-38:0) a	0.0095238	0.0667	up	670.6122	8.5
	TG(O-40:0) a	0.0095238	0.0667	up	698.6435	8.8
	TG(46:2) a	0.0057411	0.0667	down	797.6659	9.6
	TG(58:14) c	0.0057411	0.0667	down	899.6381	7.9
	TG(50:8) c	0.0057411	0.0667	down	817.6354	7.9
	TG(52:1) a	0.0089113	0.0667	down	883.7737	10.3
	TG(48:2) a	0.0095238	0.0667	up	820.7044	8.4
	TG(46:1) a	0.0095238	0.0667	up	794.6884	8.4
	TG(38:0) a	0.0095238	0.0667	up	684.6278	8.7
	TG(53:7) c	0.0095238	0.0667	down	863.6926	9.7
	TG(48:0) a	0.0095238	0.0667	down	824.7713	10.1
	TG(56:12) c	0.0095238	0.0667	up	875.6482	8.1
	TG(42:10) c	0.0095238	0.0667	up	953.6822	8.5
	TG(56:9) c	0.0095238	0.0667	up	899.7119	9.1

© 2023. Obis E et al. Published online at http://www.aginganddisease.org/EN/10.14336/AD.2023.0217

SUPPLEMENTARY DATA

**Glycerophospholipids**						
**Glycerophosphates**	PA(36:1) c	0.0057411	0.0667	down	703.5162	8.2
	PA(33:3) c	0.0089113	0.0667	down	679.4338	8.4
	PA(32:1) c	0.0095238	0.0667	down	647.4605	8.9
	PA(46:2) a	0.0095238	0.0667	up	841.7181	9
**Glycerophosphocholines**	PC(P-38:7) a	0.0095238	0.0667	up	788.5444	6.4
	PC(P-36:1) a	0.0095238	0.0667	up	772.5865	7.2
	PC(P-34:2) a	0.0095238	0.0667	up	742.5749	7.8
	PC(P-32:1) a	0.0095238	0.0667	down	760.5204	7.2
	PC(P-36:5) a	0.0095238	0.0667	up	808.5385	7.6
	PC(O-40:0) a	0.0095238	0.0667	up	832.6654	7.8
	PC(O-40:0) a	0.0095238	0.0667	up	832.6674	8
	PC(P-36:2)/PC(O-36:3) a	0.0089113	0.0667	down	770.5699	7.5
	PC(O-36:5)/PC(P-36:4) a	0.0095238	0.0667	up	748.5717	6.7
	PC(O-42:2)/PC(P-42:1) a	0.0095238	0.0667	up	820.7033	7.3
	PC(O-34:5/PC/P-34:4) a	0.0095238	0.0667	up	738.5456	7.3
	PC(P-36:2)/PC(O-36:3) a	0.0095238	0.0667	up	770.6071	7.8
	PC(O-34:1)/PC(P-34:0) a	0.0095238	0.0667	up	746.6084	7.9
	PC(O-42:6)/PC(P-42:5) a	0.0095238	0.0667	up	848.697	8.6
	PC(O-34:1)/PC(P-34:0) c	0.0095238	0.0667	up	726.5809	6.7
	PC(O-40:4)/PC(P-40:3) a	0.0095238	0.0667	down	868.5965	7.5
	PC(O-38:2)/PC(P-38:1) a	0.0095238	0.0667	up	844.5979	7.5
	PC(P-40:2)/PC(O-40:3) c	0.0095238	0.0667	up	806.6416	7.7
	PC(O-38:1)/PC(P-38:0) c	0.0095238	0.0667	up	782.6402	8.1
	PC(O-42:2)/PC-P(42:1) c	0.0095238	0.0667	up	836.6894	8.4
	PC(O-40:1)/PC(P-40:0) c	0.0095238	0.0667	up	810.674	8.4
	PC(44:12) a	0.0057411	0.0667	down	922.5686	6.9
	PC(38:5) a	0.0095238	0.0667	down	764.5238	6.7
	PC(38:6) a	0.0095238	0.0667	down	806.5722	6.8
	PC(32:1) a	0.0095238	0.0667	up	732.5566	6.9
	PC(32:0) a	0.0095238	0.0667	down	734.5734	7.4
	PC(38:4) a	0.0095238	0.0667	down	810.6035	7.5
	PC(36:2) a	0.0095238	0.0667	up	786.604	7.5
	PC(31:0) a	0.0095238	0.0667	up	720.5907	7.9
	PC(34:0) a	0.0095238	0.0667	down	762.6029	8
	PC(38:2) a	0.0095238	0.0667	up	814.6329	8
	PC(36:1) a	0.0095238	0.0667	up	788.6202	8
	PC(44:4) a	0.0095238	0.0667	up	894.6818	8.7
	PC(38:3) c	0.0095238	0.0667	up	812.6167	7.7
	PC(36:0) c	0.0095238	0.0667	up	790.6559	7.7
	PC(36:6) a	0.0095238	0.0667	down	854.5205	7
	PC(40:2) a	0.0095238	0.0667	up	918.6242	7.1
	PC(40:7) a	0.0095238	0.0667	up	876.6138	7.1
	PC(36:5) a	0.0095238	0.0667	down	872.5331	7.2
	PC(40:3) a	0.0095238	0.0667	up	932.64	7.2
	PC(40:10) c	0.0095238	0.0667	up	824.531	7.3

© 2023. Obis E et al. Published online at http://www.aginganddisease.org/EN/10.14336/AD.2023.0217

	PC(42:0) c	0.0095238	0.0667	up	854.6993	8.7
**Glycerophosphoethanolamines**	PE(P-40:6) a	0.0089113	0.0667	down	776.562	7.5
	PE(P-34:2) a	0.0095238	0.0667	up	700.5285	7.1
	PE(P-36:3) a	0.0095238	0.0667	up	726.544	7.2
	PE(P-38:4) a	0.0095238	0.0667	up	752.5611	7.5
	PE(P-34:1) a	0.0095238	0.0667	up	702.5467	7.5
	PE(P-36:2) a	0.0095238	0.0667	up	728.5626	7.6
	PE(P-38:5) a	0.0095238	0.0667	down	750.5606	7.8
	PE(P-36:1) a	0.0095238	0.0667	up	730.5772	8
	PE(P-38:2) a	0.0095238	0.0667	up	756.5921	8
	PE(P-38:1) a	0.0095238	0.0667	up	758.6074	8.4
	PE(P-40:2) a	0.0095238	0.0667	up	784.6224	8.4
	PE(P-38:3) a	0.0095238	0.0667	down	752.543	6.3
	PE(P-38:6) a	0.0095238	0.0667	down	746.5052	7
	PE(P-38:4) a	0.0095238	0.0667	up	750.5377	7.5
	PE(P-40:6) a	0.0095238	0.0667	down	774.5365	7.5
	PE(P-34:1) a	0.0095238	0.0667	up	700.5222	7.5
	PE(P-36:2) a	0.0095238	0.0667	up	726.5369	7.6
	PE(P-40:4) a	0.0095238	0.0667	up	778.5673	7.9
	PE(P-36:1) a	0.0095238	0.0667	up	728.5525	8
	PE(P-38:2) a	0.0095238	0.0667	up	754.5675	8
	PE(P-42:0) c	0.0095238	0.0667	up	796.6577	8.2
	PE(P-38:1) a	0.0095238	0.0667	up	756.5823	8.4
	PE(P-40:2) a	0.0095238	0.0667	up	782.5973	8.4
	PE(P-40:1) a	0.0095238	0.0667	up	784.6124	8.8
	PE(P-36:2)/PE(O-36:3) a	0.0095238	0.0667	up	726.5811	7.2
	PE(P-38:2)/PE(O-38:3) a	0.0095238	0.0667	up	754.6103	7.7
	PE(40:5) a	0.0057411	0.0667	down	794.5712	7.5
	PE(44:10) c	0.0057411	0.0667	down	870.5166	6.8
	PE-NMe(42:6)a	0.0057411	0.0667	down	832.5379	7.6
	PE(36:1) a	0.0095238	0.0667	up	746.571	7.2
	PE(40:6) a	0.0095238	0.0667	down	792.5569	7.2
	PE(36:2) a	0.0095238	0.0667	up	744.5562	7.3
	PE(38:4) a	0.0095238	0.0667	down	768.5567	7.3
	PE(34:2) a	0.0095238	0.0667	up	716.56	7.7
	PE(40:2) a	0.0095238	0.0667	up	800.6169	7.7
	PE(40:7) a	0.0095238	0.0667	up	788.5371	6.8
	PE(36:2) a	0.0095238	0.0667	up	742.5733	7
	PE(32:0) a	0.0095238	0.0667	up	672.4894	7
	PE(40:6) a	0.0095238	0.0667	down	790.5333	7.2
	PE(36:2) a	0.0095238	0.0667	up	742.5316	7.3
	PE(38:4) a	0.0095238	0.0667	down	766.5311	7.3
	PE(40:5) a	0.0095238	0.0667	down	792.5643	7.4
	PE(44:3) b	0.0095238	0.0667	down	900.5848	7.5
	PE-NMe(38:3) a	0.0095238	0.0667	up	782.5222	7.5
	PE(40:4) a	0.0095238	0.0667	down	794.5611	7.7
	PE(40:2) a	0.0095238	0.0667	up	798.6372	7.7
	PE(34:2) a	0.0095238	0.0667	up	714.5364	7.8
	PE(40:0) a	0.0095238	0.0667	up	802.5869	7.9
	PE(40:2) a	0.0095238	0.0667	up	798.638	7.9
	PE(38:1) a	0.0095238	0.0667	up	772.5768	8.1
	PE(36:2) a	0.0095238	0.0667	up	742.5653	8.2
	PE(44:0) c	0.0095238	0.0667	up	840.6849	8.5
	PE(46:0) b	0.0095238	0.0667	up	922.6838	8.5
	PE-NMe2(44:11) c	0.0095238	0.0667	down	864.5665	6.8
	LPE(18:0) a	0.0057411	0.0667	down	480.3047	0.9
	LPE(18:1) a	0.0095238	0.0667	up	480.311	0.9

© 2023. Obis E et al. Published online at http://www.aginganddisease.org/EN/10.14336/AD.2023.0217

SUPPLEMENTARY DATA

	LPE(18:1) a	0.0095238	0.0667	up	478.2892	0.9
	LPE(22:4) a	0.0095238	0.0667	up	528.3035	2.7
**Glycerophosphoglycerols**	PG(39:8) c	0.0095238	0.0667	down	822.4828	6
	PG(28:2) a	0.0095238	0.0667	down	663.4561	7.8
	PG(44:2) a	0.0095238	0.0667	up	885.667	8.2
	PG(44:0) c	0.0095238	0.0667	up	889.6632	8.3
	PG(44:2) a	0.0095238	0.0667	up	885.6969	8.8
	PG(44:1) c	0.0095238	0.0667	up	887.6475	7.9
	BMP(34:2) a	0.0095238	0.0667	down	764.5774	7.8
**Glycerophosphoglycero-phosphoglycerols**	CL(78:11) a	0.0057411	0.0667	down	762.5007	6.7
	CL(78:9) b	0.0095238	0.0667	up	764.551	7.7
	CL(74:5) b	0.0095238	0.0667	up	740.5514	7.8
**Glycerophosphoserines**	PS(P-42:7) a	0.0057411	0.0667	down	844.5004	6.7
	PS(P-40:1) a	0.0095238	0.0667	up	828.6028	7.9
	PS(42:9) c	0.0057411	0.0667	down	856.5023	6.4
	PS(44:5) c	0.0057411	0.0667	down	892.5978	7.4
	PS(40:6) a	0.0095238	0.0667	down	836.5444	6.4
	PS(36:1) a	0.0095238	0.0667	up	790.5607	6.8
	PS(40:6) a	0.0095238	0.0667	down	834.5214	6.4
	PS(36:2) a	0.0095238	0.0667	up	786.5197	6.4
	PS(40:4) a	0.0095238	0.0667	up	838.5484	6.8
	PS(46:6) c	0.0095238	0.0667	up	900.6142	6.8
	PS(40:3) a	0.0095238	0.0667	down	840.5656	6.9
	PS(40:9) c	0.0095238	0.0667	down	828.5043	7
	PS(44:4) c	0.0095238	0.0667	up	902.6303	7.1
	PS(46:2) c	0.0095238	0.0667	up	908.6685	8.3
**Sphingolipids**						
**Acidic glycosphingolipids**	Sulfatide(36:1) a	0.0095238	0.0667	up	806.5378	6
	Sulfatide(43:1) a	0.0095238	0.0667	up	904.611	6.8
	Sulfatide(42:2) a	0.0095238	0.0667	up	888.6163	6.9
	Sulfatide(42:0) a	0.0095238	0.0667	up	892.6085	7.1
	Sulfatide(43:0) a	0.0095238	0.0667	up	906.625	7.3
	Sulfatide(44:2) a	0.0095238	0.0667	up	916.6456	7.3
	Sulfatide(42:1) a	0.0095238	0.0667	up	890.63	7.3
	Sulfatide(40:4) a	0.0095238	0.0667	down	856.5331	7.5
	Sulfatide(43:1) a	0.0095238	0.0667	up	904.645	7.5
**Ceramides**	CerP(d40:1) a	0.0095238	0.0667	up	684.5787	6.9
	OxCer(48:1) a	0.0095238	0.0667	down	748.7278	7.2
	Cer(d42:3) a	0.0095238	0.0667	up	646.6169	7.9
	Cer(d40:2) a	0.0095238	0.0667	up	620.5983	7.9
	OxCer(42:2) a	0.0095238	0.0667	up	630.6205	8
	Cer(d40:6) c	0.0095238	0.0667	down	612.5028	8.1
	Cer(d43:3) a	0.0095238	0.0667	up	660.6303	8.1
	Cer(d41:2) a	0.0095238	0.0667	up	634.615	8.1
	Cer(d38:1) a	0.0095238	0.0667	down	594.5836	8.1
	Cer(d44:3) a	0.0095238	0.0667	up	674.6448	8.3
	Cer(d42:2) a	0.0095238	0.0667	up	630.6214	8.5
	Cer(d42:4) a	0.0095238	0.0667	up	644.6348	8.7
	Cer(d40:3) a	0.0095238	0.0667	up	618.6196	8.7
	Cer(d43:4) c	0.0095238	0.0667	up	658.6501	8.9
	Cer(d44:4) a	0.0095238	0.0667	up	672.6275	8.9
	Cer(d42:1) a	0.0095238	0.0667	up	632.6347	8.9
	Cer(d16:0) c	0.0095238	0.0667	down	332.2617	2.7
	Cer(d48:2) a	0.0095238	0.0667	down	746.7042	7.2
	Cer(d36:1) a	0.0095238	0.0667	up	564.5301	7.7
	Cer(d42:3) c	0.0095238	0.0667	up	644.5902	8.2
	Cer(d42:2) a	0.0095238	0.0667	up	692.6111	8.5
	Cer(d42:2) a	0.0095238	0.0667	up	646.6062	8.5
	Cer(d40:1) c	0.0095238	0.0667	up	620.5902	8.5

© 2023. Obis E et al. Published online at http://www.aginganddisease.org/EN/10.14336/AD.2023.0217

	Cer(d43:3) a	0.0095238	0.0667	up	674.6375	8.9
	Cer(d42:1) c	0.0095238	0.0667	up	648.6215	8.9
	Cer(d43:1) c	0.0095238	0.0667	up	662.6357	9
**Neutral glycosphingolipids**	HexCer(d36:2) a	0.0095238	0.0667	up	726.5889	7
	HexCer(d42:4) a	0.0095238	0.0667	up	806.609	7.9
	HexCer(d40:4) a	0.0095238	0.0667	up	778.6568	7.9
	HexCer(d42:3) a	0.0095238	0.0667	up	808.6695	7.9
	HexCer(d40:2) a	0.0095238	0.0667	up	782.6517	7.9
	HexCer(d42:2) b	0.0095238	0.0667	up	792.6754	8
	HexCer(d40:1) a	0.0095238	0.0667	up	766.6576	8.1
	HexCer(d43:3) a	0.0095238	0.0667	up	822.684	8.1
	HexCer(d41:2) a	0.0095238	0.0667	up	796.6687	8.1
	HexCer(d42:3) a	0.0095238	0.0667	up	808.6249	8.2
	HexCer(d40:3) a	0.0095238	0.0667	up	780.6729	8.2
	HexCer(d44:3) a	0.0095238	0.0667	up	836.6953	8.3
	HexCer(d43:2) a	0.0095238	0.0667	up	824.7002	8.5
	HexCer(d42:4) a	0.0095238	0.0667	up	806.69	8.2
	HexCer(d42:3) a	0.0095238	0.0667	up	808.7043	8.6
	HexCer(t40:2) a	0.0095238	0.0667	down	812.6734	6.2
	HexCer(d36:2) c	0.0095238	0.0667	up	886.6001	6.6
	HexCer(t33:3) a	0.0095238	0.0667	up	712.5196	7.4
	HexCer(d42:0) a	0.0095238	0.0667	up	812.6537	7.9
	HexCer(d43:1) a	0.0095238	0.0667	up	824.6537	7.9
	HexCer(d42:2) a	0.0095238	0.0667	up	808.6597	8.1
	HexCer(t42:3) a	0.0095238	0.0667	up	838.6688	8.1
	HexCer(d42:0) a	0.0095238	0.0667	up	812.6538	8.1
	HexCer(t43:3) a	0.0095238	0.0667	up	852.6833	8.3
	HexCer(d43:0) a	0.0095238	0.0667	up	826.6701	8.3
	HexCer(d43:1) c	0.0095238	0.0667	up	824.6892	8.6
**Phosphosphingolipids**	SM(d34:1) a	0.0095238	0.0667	up	703.5768	6.7
	SM(d36:2) a	0.0095238	0.0667	up	729.5938	6.8
	SM(d35:1) a	0.0095238	0.0667	up	717.5907	7
	SM(d40:2) a	0.0095238	0.0667	up	785.6552	7.9
	SM(d38:1) a	0.0095238	0.0667	down	759.6399	7.8
	SM(d43:2) a	0.0095238	0.0667	up	827.7037	8.7
	SM(d41:1) a	0.0095238	0.0667	up	801.685	8.7
	SM(d42:1) a	0.0095238	0.0667	up	815.702	9.1
	SM(d43:2) a	0.0095238	0.0667	up	871.6536	8
	SM(d43:2) a	0.0095238	0.0667	up	871.6809	8.5
**Sterol Lipids**						
**Sterols**	CE(15:0) c	0.0095238	0.0667	down	645.5232	8.6
	OxCE(18:1) c	0.0095238	0.0667	down	687.5694	9

Lipidomic features with Mann-Whitney U test p-value<0.05. We have classified the lipid species according to the confidence of the identification: a) exact mass, retention time and MS/MS spectrum (high reliability), b) exact mass, retention time and MS/MS spectrum (medium reliability), c) exact mass and retention time. FDR: False Discovery Rate. FDR p-values were corrected for 2,048 tests.

© 2023. Obis E et al. Published online at http://www.aginganddisease.org/EN/10.14336/AD.2023.0217

SUPPLEMENTARY DATA

Supplementary Table 4. Unidentified significant distinctive lipidomic features for white and grey matter in brain tissue.

Class	Compound	Mann-Whitney p-value	Mann-Whitney FDR p-value	Regulation (WM vs GM)	m/z value	Retention time
**Unknown**						
**Unknown**	398.19_0.86392	0.0057411	0.0667	down	399.1973	0.9
	677.5246_8.0952	0.0057411	0.0667	down	678.5319	8.1
	668.5212_8.6017	0.0057411	0.0667	down	669.5285	8.6
	280.2378_3.0610	0.0057411	0.0667	down	279.2305	3.1
	565.6891_7.1682	0.0089113	0.0667	down	566.6964	7.2
	634.5336_7.4776	0.0089113	0.0667	down	635.5409	7.5
	238.1211_0.8675	0.0095238	0.0667	down	239.1284	0.9
	796.1554_5.6956	0.0095238	0.0667	down	797.1627	5.7
	780.1825_5.9419	0.0095238	0.0667	down	781.1898	5.9
	560.5174_7.5109	0.0095238	0.0667	up	561.5247	7.5
	514.4072_7.5952	0.0095238	0.0667	down	515.4145	7.6
	527.4968_7.6575	0.0095238	0.0667	down	528.5041	7.7
	735.5375_7.7941	0.0095238	0.0667	down	736.5448	7.8
	541.5115_7.8739	0.0095238	0.0667	down	542.5188	7.9
	317.2983_8.0923	0.0095238	0.0667	down	318.3056	8.1
	492.4916_8.0789	0.0095238	0.0667	down	493.4989	8.1
	312.2668_8.1176	0.0095238	0.0667	up	313.2741	8.1
	338.2824_8.1365	0.0095238	0.0667	up	339.2897	8.1
	1314.2874_8.382	0.0095238	0.0667	up	1315.2947	8.4
	1242.2812_8.378	0.0095238	0.0667	up	1243.2885	8.4
	340.298_8.50841	0.0095238	0.0667	up	341.3053	8.5
	338.2826_8.5110	0.0095238	0.0667	up	339.2899	8.5
	872.2598_8.8562	0.0095238	0.0667	down	873.2671	8.9
	242.1491_0.9088	0.0095238	0.0667	down	241.1418	0.9
	310.2843_4.5641	0.0095238	0.0667	up	309.2770	4.6
	366.3459_6.2096	0.0095238	0.0667	up	365.3386	6.2
	394.3772_6.9073	0.0095238	0.0667	up	393.3699	6.9
	1164.2555_7.911	0.0095238	0.0667	down	1163.2482	7.9
	1238.2736_8.184	0.0095238	0.0667	down	1237.2663	8.2
	823.6816_8.2384	0.0095238	0.0667	up	822.6743	8.2
	633.5969_8.3553	0.0095238	0.0667	up	632.5896	8.4
	1386.309_8.5942	0.0095238	0.0667	down	1385.3017	8.6
	661.6291_8.7003	0.0095238	0.0667	up	660.6218	8.7

Lipidomic features with Mann-Whitney U test p-value<0.01, non-identified by MS, RT and MS/MS spectra classified as Unknowns. FDR: False Discovery Rate. FDR p-values were corrected for 2,048 tests.

© 2023. Obis E et al. Published online at http://www.aginganddisease.org/EN/10.14336/AD.2023.0217

SUPPLEMENTARY DATA

Supplementary Table 5. Unidentified significant distinctive lipidomic features in grey matter during AD progression.

Class	Compound	Kruskal-Wallis p-value	Kruskal-Wallis FDR p-value	Post-hoc (Dunn's Test)	m/z value	Retention time
**Unknown**						
**Unknown**	1077.2719_7.635	0.010448	0.845	A-C A-D B-C C-D	1078.2792	7.64
	1080.2548_7.635	0.031718	0.845	A-C B-C	1081.2621	7.64
	1092.2303_7.625	0.021924	0.845	A-D B-D C-D	1093.2376	7.63
	110.0361_0.8535	0.016152	0.845	A-B A-C A-D	111.0434	0.85
	128.046_0.79454	0.02328	0.845	A-B A-C B-D	127.0387	0.79
	211.1594_0.9228	0.038388	0.845	A-B	212.1667	0.92
	214.0206_0.7680	0.024433	0.845	A-D B-D C-D	213.0133	0.77
	239.2629_10.549	0.020236	0.845	A-C B-C C-D	240.2702	10.55
	302.1642_2.2336	0.029211	0.845	B-D C-D	301.1569	2.23
	308.2141_4.6456	0.048831	0.845	B-C C-D	307.2068	4.65
	329.3286_3.2113	0.009155	0.845	A-B A-D B-C C-D	330.3359	3.21
	335.3168_0.9753	0.028794	0.845	A-C B-C C-D	336.3241	0.98
	340.093_0.84219	0.020709	0.845	A-D B-D C-D	341.1003	0.84
	340.1492_0.8236	0.043647	0.845	A-C B-C C-D	341.1565	0.82
	346.2181_7.5950	0.04852	0.845	A-D B-C B-D	347.2254	7.6
	360.1745_0.8947	0.022657	0.845	A-B A-C A-D	359.1672	0.89
	360.3367_0.9784	0.043616	0.845	A-D B-D	361.344	0.98
	398.19_0.86392	0.029269	0.845	A-C B-C C-D	399.1973	0.86
	418.309_5.52832	0.017704	0.845	A-B B-D	419.3163	5.53
	481.3119_3.4994	0.031466	0.845	A-B A-C A-D	480.3046	3.5
	60.0204_0.86653	0.023184	0.845	A-C C-D	59.0131	0.87
	612.1748_7.7724	0.04124	0.845	A-C B-C C-D	613.1821	7.77
	851.9602_7.1495	0.045484	0.845	A-D	850.9529	7.15
	965.22_9.025290	0.016541	0.845	A-D B-C C-D	966.2273	9.03
	997.283_7.26389	0.04957	0.845	B-D C-D	998.2903	7.26

Lipidomic features with Kruskal-Wallis test p-value < 0.05, non-identified by MS, RT and MS/MS spectra classified as Unknowns. FDR: False Discovery Rate. FDR p-values were corrected for 2,048 tests.

© 2023. Obis E et al. Published online at http://www.aginganddisease.org/EN/10.14336/AD.2023.0217

SUPPLEMENTARY DATA

Supplementary Table 6. Unidentified significant distinctive lipidomic features in white matter during AD progression.

Class	Compound	Kruskal-Wallis p-value	Kruskal-Wallis FDR p-value	Post-hoc (Dunn's Test)	m/z value	Retention time
**Unknown**						
**Unknown**	1053.2917_9.502	0.033862	0.801	A-C B-C C-D	1054.299	9.5
	1094.247_7.9067	0.022435	0.801	B-C B-D	1095.2543	7.91
	124.0514_0.8604	0.040452	0.801	A-B A-C A-D	123.0441	0.86
	126.0676_0.8716	0.035964	0.801	A-B A-C A-D	127.0749	0.87
	166.1354_0.9250	0.025681	0.801	A-B B-D C-D	167.1427	0.93
	172.1447_0.9338	0.045498	0.801	A-D B-D C-D	171.1374	0.93
	199.1934_1.8807	0.037332	0.801	A-C B-C	200.2007	1.88
	208.1917_0.9568	0.020253	0.801	A-D B-D C-D	209.199	0.96
	210.1597_0.9174	0.023644	0.801	B-C B-D	209.1524	0.92
	238.2174_10.554	0.0065421	0.801	A-B B-C C-D	239.2247	10.55
	240.1641_0.9626	0.0066994	0.801	A-B B-C B-D	239.1568	0.96
	242.1155_0.8251	0.031692	0.801	A-D B-C C-D	243.1228	0.83
	250.1576_0.9329	0.014293	0.801	A-C B-C C-D	251.1649	0.93
	254.1886_0.9288	0.026092	0.801	A-C C-D	255.1959	0.93
	286.0828_0.8305	0.039072	0.801	A-C B-C	287.0901	0.83
	324.154_0.86864	0.038458	0.801	A-D B-C C-D	325.1613	0.87
	360.3337_0.9602	0.045854	0.801	A-B B-C	359.3264	0.96
	388.2972_8.4307	0.01834	0.801	A-C B-C C-D	389.3045	8.43
	406.2708_0.9220	0.025195	0.801	A-B A-C C-D	405.2635	0.92
	446.2244_6.7663	0.005119	0.801	A-D B-D C-D	445.2171	6.77
	552.3831_6.3107	0.028744	0.801	A-C B-C C-D	553.3904	6.31
	592.5909_8.4160	0.0098526	0.801	A-C B-C C-D	593.5982	8.42
	657.7232_7.1611	0.047273	0.801	A-B A-C A-D	656.7159	7.16
	667.0593_0.9134	0.0038145	0.801	A-B A-C A-D B-C C-D	666.052	0.91
	685.5312_0.9721	0.032858	0.801	A-C B-D C-D	686.5385	0.97
	706.164_5.34443	0.040145	0.801	A-B A-D B-C	707.1713	5.34
	720.6389_7.5765	0.030618	0.801	A-C B-C	721.6462	7.58
	744.2015_0.9867	0.041729	0.801	B-C B-D	743.1942	0.99
	762.1933_6.9904	0.039731	0.801	A-B A-C B-D C-D	763.2006	6.99
	771.2663_8.8725	0.033477	0.801	A-C C-D	772.2736	8.87
	822.2932_5.4247	0.049824	0.801	A-C C-D	823.3005	5.42
	845.6082_0.9154	0.011592	0.801	A-B A-C B-D	846.6155	0.92
	854.2024_6.4596	0.035044	0.801	A-C A-D B-C	855.2097	6.46

Lipidomic features with Kruskal-Wallis test p-value < 0.05, non-identified by MS, RT and MS/MS spectra classified as Unknowns. FDR: False Discovery Rate. FDR p-values were corrected for 2,048 tests.

© 2023. Obis E et al. Published online at http://www.aginganddisease.org/EN/10.14336/AD.2023.0217
